# *CircRPAP2* regulates the alternative splicing of PTK2 by binding to SRSF1 in breast cancer

**DOI:** 10.1038/s41420-022-00965-y

**Published:** 2022-04-02

**Authors:** Yunhe Yu, Lin Fang

**Affiliations:** 1 Department of Breast and Thyroid Surgery, Shanghai Tenth People’s Hospital, School of Medicine, Tongji University, Shanghai, 200072 China; 2 School of Medicine, Tongji University, Shanghai, 200092 China

**Keywords:** Breast cancer, Non-coding RNAs

## Abstract

Breast cancer is the most commonly diagnosed malignant tumor and the second-highest cause of cancer-related deaths in women worldwide. Circular RNAs (circRNAs) are associated with the development of numerous cancers, including breast cancer. Here, we present the first report that *circRPAP2* (hsa_circ_0000091) is downregulated in breast cancer tissue samples and cell lines. Furthermore, the expression level of *circRPAP2* in breast cancer tissues was correlated with axillary lymph node metastasis and TNM stage. Biological function studies demonstrated that *circRPAP2* inhibited the proliferation and migration of breast cancer in vivo and in vitro. The mechanistic evaluation indicated that *circRPAP2* can bind to the oncoprotein SRSF1, likely competing with the binding between SRSF1 and *PTK2* pre-mRNA, thereby attenuating SRSF1-mediated alternate splicing of PTK2, an effector of SRSF1 oncogenic activity, resulting in the reduction of PTK2 mRNA and protein expression. Overall, our findings suggest that *circRPAP2* plays a tumor suppressor role and may serve as a biomarker in breast cancer. In addition, the identification of the *circRPAP2*/SRSF1*/*PTK2 axis provides new insights into the pathogenesis of breast cancer and highlights a novel target for the development of oncotherapeutics.

## Introduction

Breast cancer (BC) is the most commonly diagnosed malignant tumor and the second leading cause of cancer-related deaths in women worldwide [1]. Although notable progress with regard to early diagnosis and comprehensive treatment strategies has improved the prognosis of patients with BC, the morbidity and mortality are still increasing [2]. In addition, the incidence of metastasis has also surged, accounting for approximately 90% of BC-related deaths [3]. Therefore, identifying effective biomarkers for BC diagnosis and prognosis along with their associated molecular mechanisms is vital for enhancing patient outcome.

Circular RNA (circRNA) is a type of noncoding RNA produced by back-splicing. The loop structures of circRNAs render them more stable than linear RNAs terminated with 5′ caps and 3′ tails. circRNAs have various biological functions in cancers, such as serving as competing endogenous RNAs (ceRNAs) or microRNA (miRNA) sponges [4–6], interacting with proteins [7–9], regulating gene transcription [10, 11], and acting as a template for translation [12–14]. Furthermore, it has been confirmed that cancer cell-derived circRNAs in the circulation can be assayed for cancer detection [15, 16] and can accurately reflect the changes in cancer cells during tumor progression and metastasis [17].

In particular, *circRPAP2* (hsa_circ_0000091, derived from the host gene *RPAP2*) is differentially expressed in BC plasma; [18] however, its biological functions and mechanisms in BC remained unknown. Herein, we evaluated the correlation between *circRPAP2* expression in BC tissues and clinical features of patients. Furthermore, we performed functional and mechanistic studies in BC cells and a female mouse xenograft model to clarify the role of *circRPAP2* in BC and its potential as a novel target for oncotherapeutics. This is the first study to demonstrate that *circRPAP2* inhibits the proliferation and migration of BC in vivo and in vitro by binding with the oncoprotein SRSF1 to regulate the alternative splicing of PTK2. Our results revealed a new regulatory mechanism for circRNA in BC and provided evidence that *circRPAP2* may constitute a biomarker for BC.

## Results

### Characteristics of *circRPAP2* in BC

**Fig. 1:**
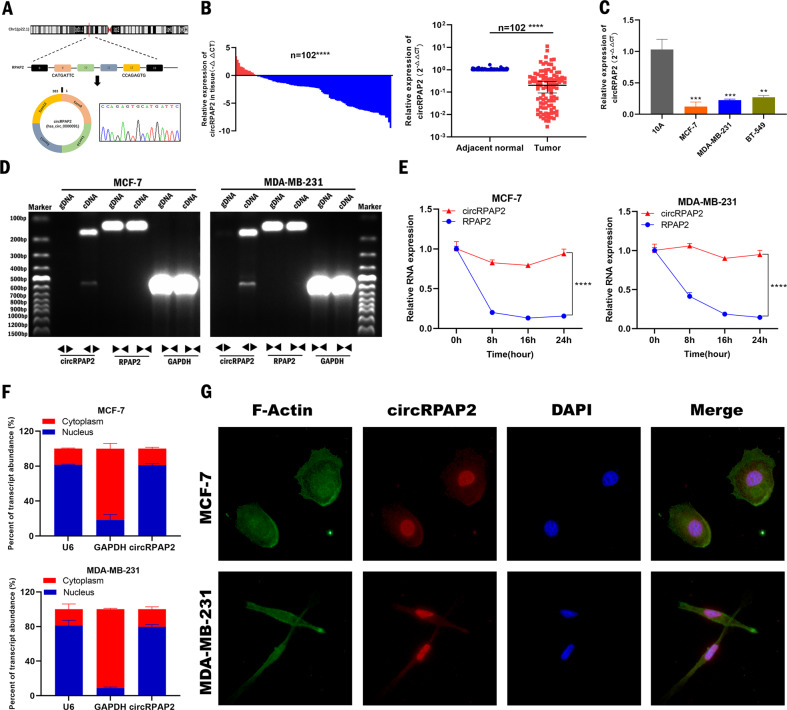
Characteristics of *circRPAP2* in BC. **A** The structure of *circRPAP2* (hsa_circ_0000091) and the splicing junction of *circRPAP2* was verified by Sanger sequencing **B** Level of *circRPAP2* in BC tissues compared with that in the adjacent normal tissues (*n* = 102) as determined by qRT-PCR. **C** Level of *circRPAP2* in BC cell lines. **D** and **E** The existence of *circRPAP2* in BC cells was verified using agarose gel electrophoresis and actinomycin D assays. **F** Localization of *circRPAP2* in BC cells as detected using the cytoplasm–nucleus separation assay. **G** Localization of *circRPAP2* in BC cells as detected by RNA FISH. (*P < 0.05, **P < 0.01, ***P < 0.001, ****P < 0.0001).

*CircRPAP2*, also known as hsa_circ_0000091 (chr1:92798947–92846430 (GRCh38.p13)), is a 383 nt-long circRNA formed by circularization of exons 9–12 of *RPAP2*, and the splicing junction of *circRPAP2* was verified by Sanger sequencing (Fig. 1A). We analyzed the expression of *circRPAP2* by quantitative reverse transcription-polymerase chain reaction (qRT-PCR) in 102 pairs of BC and adjacent normal tissues. Our results suggested that the expression of *circRPAP2* was significantly reduced in BC tissues (89/102, 87.25%, *p* < 0.0001) compared with that in the adjacent normal tissues (Fig. 1B). The expression of *circRPAP2* was also significantly decreased in BC cell lines (MCF-7, MDA-MB-231, and BT-549) compared with that in the normal breast epithelial cell line MCF-10A (Fig. 1C). In addition, relationships between *circRPAP2* expression levels and clinical features of 102 patients with BC were analyzed. The expression level of *circRPAP2* was associated with axillary lymph node metastasis and TNM stage; however, no significant associations with age, tumor grade, tumor size, Ki-67, or molecular subtypes were observed (Table 1).

**Table 1:** Relationships between *circRPAP2* expression and clinical features of patients with BC.

Features	Total	*circRPAP2*		*P* value
	(*n* = 102)	Low	High	
Age				
<50	19	5	14	0.171
≥50	83	36	47	
Grades				
I	12	4	8	0.585
II	46	21	25	
III	44	16	28	
Tumor size				
Tis	11	3	8	0.505
T1	42	18	24	
T2	47	20	27	
T3	2	0	2	
ALN status				
ALN−	77	25	52	0.005**
ALN +	25	16	9	
Stage				
I-II	84	29	55	0.012*
III	18	12	6	
Ki-67				
≤30	33	38	71	0.050
>30	8	23	31	
Molecular subtype				
Luminal A	27	14	13	0.290
Luminal B	46	15	31	
HER2 overexpression	13	4	9	
TNBC	16	8	8	

The TNM Stage System is based on the tumor (T), the extent of spread to the lymph nodes (N), and the presence of metastasis (M). ALN: axillary lymph node.

*P < 0.05, **P < 0.01 were considered statistically significant.

To confirm that *circRPAP2* was circular in form, we examined the gDNA and cDNA PCR products amplified with primers specific for *circRPAP2*, the host gene *RPAP2*, and reference gene *GAPDH* using 2% agarose gel electrophoresis. Specific bands of *circRPAP2* were observed only with the cDNA PCR products (Fig. 1D). Subsequently, actinomycin D assays were conducted (Fig. 1E) to further verify that *circRPAP2* constituted a stable circular structure that could not easily be degraded. Cytoplasm–nucleus separation assays and fluorescence in situ hybridization (FISH) were used to explore the cellular distribution of *circRPAP2*. The expression level of *circRPAP2* was examined by qRT-PCR in the cytoplasmic and nuclear fractions of MCF-7 and MDA-MB-231 cell lines. Subcellular fractionation quantitatively demonstrated that *circRPAP2* was enriched in the nucleus (Fig. 1F). RNA FISH experiments also revealed that *circRPAP2* was mainly localized in the nucleus of BC cell lines (Fig. 1G).

### *CircRPAP2* inhibits BC cell proliferation and migration in vitro

**Fig. 2:**
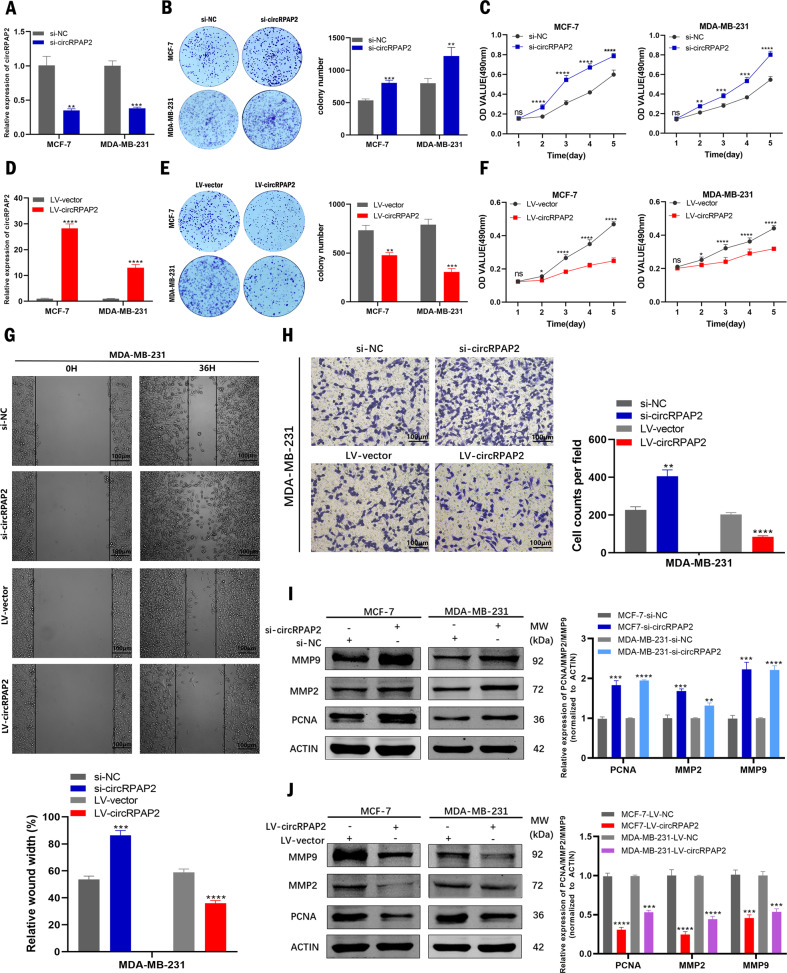
*CircRPAP2* inhibits BC cell proliferation and migration in vitro. **A** and **D** Relative expression levels of *circRPAP2* were confirmed by qRT-PCR in BC cells transfected with si-NC, si-*circRPAP2* (**A**), LV–vector, or *LV-circRPAP2*(D). **B** and **E** Effect of *si-circRPAP2* (**B**) and *LV-circRPAP2* (**E**) on BC cell proliferation as determined by colony formation assays. **C** and **F** Effect of *si-circRPAP2* (**C**) and *LV-circRPAP2* (**F**) on BC cell proliferation as determined by MTT assays. **G** Results of wound healing assays performed using MDA-MB-231 cells. **H** Results of the Transwell migration assays performed using MDA-MB-231 cells. **I** and **J** Effect of *si-circRPAP2* (**I**) and *LV-circRPAP2* (**J**) on BC cell proliferation and migration as determined by western blotting. (*P < 0.05, **P < 0.01, ***P < 0.001, ****P < 0.0001).

To investigate whether *circRPAP2* affects the biological processes of BC cells, the expression of *circRPAP2* in MCF-7 and MDA-MB-231 cell lines was downregulated using individual-specific siRNAs (*si-circRPAP2, si2-circRPAP2*, or *si3-circRPAP2*); si-NC was used as a control. We also stably overexpressed *circRPAP2* in MCF-7 and MDA-MB-231 cell lines; the LV-vector was used as a control. The expression level of *circRPAP2* was verified by qRT-PCR. The results showed that *si-circRPAP2* exhibited high interference efficiency and that *LV-circRPAP2* overexpressed *circRPAP2* more than 15-fold (Fig. 2A and D). Colony formation and MTT assays, used to examine the effect of *circRPAP2* on BC proliferation, indicated that *circRPAP2* depletion promoted cell proliferation in MCF-7 and MDA-MB-231 cells (Fig. 2B and C), whereas cell proliferation was significantly inhibited by *circRPAP2* overexpression (Fig. 2E and F). To explore the role of *circRPAP2* in BC migration, wound healing and Transwell migration assays were conducted using MDA-MB-231 cells. The results demonstrated that cell migration was increased by *circRPAP2* depletion but decreased by *circRPAP2* overexpression (Fig. 2G and H). Western blotting confirmed that the expression of the proliferation marker PCNA and migration markers MMP2 and MMP9 was promoted by *si-circRPAP2* (Fig. 2I). In contrast, the expression of PCNA, MMP2, and MMP9 was inhibited by *LV-circRPAP2* (Fig. 2J). In comparison, *si2-circRPAP2* and *si3-circRPAP2* exhibited minor interference ability (Figure S1a). Consistent with this, the results of the MMT assays and western blotting indicated that BC cell biological processes did not significantly change following transfection of *si2-circRPAP2* or *si3-circRPAP2* (Figure S1b–f). Consequently, only *si-circRPAP2* was used in the subsequent experiments.

### *CircRPAP2* binds to the oncoprotein SRSF1 in the nucleus

**Fig. 3:**
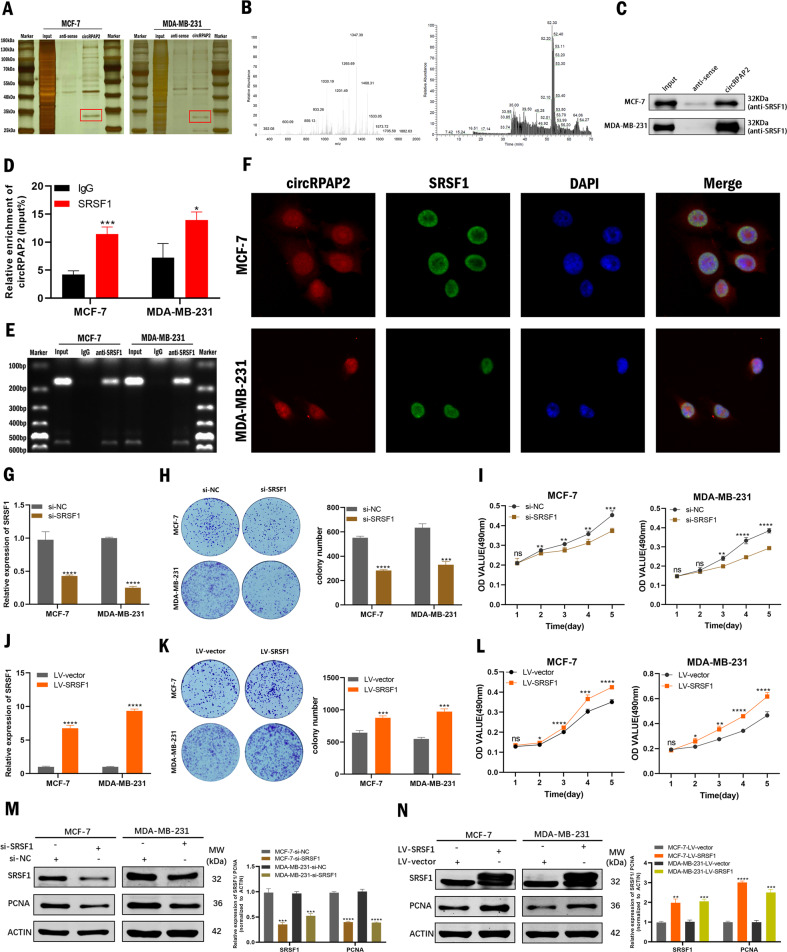
*CircRPAP2* binds to the oncoprotein *SRSF1* in the nucleus. **A** Silver staining of *circRPAP2* pulldown products. Red squares indicate bands differing between the sense and anti-sense lanes. **B** Protein mass spectrometry analysis of the different bands. **C–E** Results of RNA pulldown and RIP assays to identify binding between *circRPAP2* and SRSF1. **F** Combination of FISH and IF assays indicates *circRPAP2* and SRSF1 are co-localized, mainly in the cell nucleus. **G** and **J** Relative expression of *SRSF1* was confirmed by qRT-PCR in BC cells transfected with si-NC, si-*SRSF1*(**G**), LV–vector, or *LV-SRSF1* (**J**). **H** and **K** Effect of *si-SRSF1* (**H**) and *LV-SRSF1* (**K**) on BC cell proliferation as determined by colony formation assays. **I** and **L** Effect of *si-SRSF1* (**I**) and *LV-SRSF1* (**L**) on BC cell proliferation as determined by MTT assays. **M** and **N** Effect of *si-SRSF1* (**M**) and *LV-SRSF1* (**N**) on BC cell proliferation in BC cells as determined by western blotting. (*P < 0.05, **P < 0.01, ***P < 0.001, ****P < 0.0001).

Considering that *circRPAP2* was mainly located in the nucleus, we evaluated whether the mechanism of *circRPAP2* in BC involved interaction with an RNA binding protein (RBP). We designed a specific biotin-labeled *circRPAP2* probe to perform endogenous RNA pulldown assays in BC cells. Silver staining highlighted the enrichment of several protein bands in the *circRPAP2* probe group compared to those in the anti-sense group (control group) (Fig. 3A). The most stable and markedly differentially expressed protein band, with a molecular weight of approximately 35 kDa, was selected for protein mass spectrometry analysis. This result implied that the protein most likely represented arginine-rich splicing factor 1 (SRSF1) in the ranking list of recognized proteins (Fig. 3B), which was confirmed by subsequent western blotting using an anti-SRSF1 antibody (Fig. 3C). We then collected the RNA pulled down by the anti-SRSF1 antibody via RNA immunoprecipitation (RIP) assay. Both qRT-PCR and agarose gel electrophoresis assay revealed the presence of abundant *circRPAP2* compared to IgG (Fig. 3D and E). In addition, FISH and immunofluorescence (IF) assays indicated the co-localization of *circRPAP2* and SRSF1 mainly in the cell nucleus (Fig. 3F), consistent with *circRPAP2*–SRSF1 binding.

SRSF1 (previously known as SF2/ASF) is a prototypical SR protein of molecular weight 32 kDa that has been reported to function as an oncoprotein [19]. *SRSF1* is upregulated in human breast tumors, and its overexpression promotes the transformation of mammary cells [20]. Consistent with this, in the present study, we demonstrated that knockdown of SRSF1 (Fig. 3G) inhibited cell proliferation, whereas overexpression (Fig. 3J) of SRSF1 promoted cell proliferation in BC cells as determined using the colony formation assays (Fig. 3H and K), MTT assays (Fig. 3I and L), and western blotting (Fig. 3M and N). In addition, we explored whether SRSF1 affected the biogenesis of *circRPAP2*, and the results are shown in Figure S2. The expression of *circRPAP2* and *RPAP2* were not regulated by SRSF1.

### *CircRPAP2* regulates the alternative splicing of PTK2 by binding to SRSF1

**Fig. 4:**
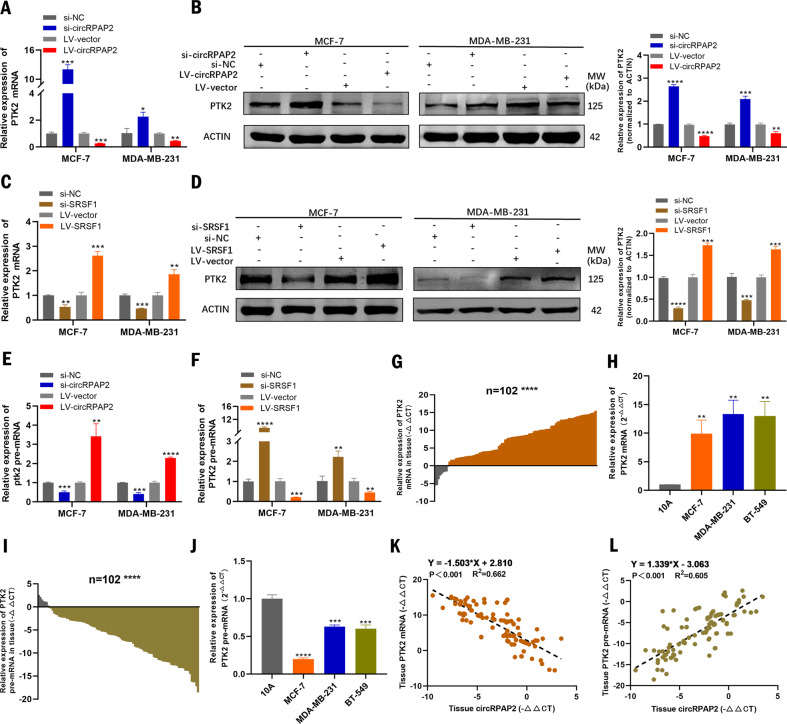
*CircRPAP2* and SRSF1 are involved in the alternative splicing of *PTK2*. **A** and **B** Expression levels of PTK2 mRNA and protein were detected by qRT-PCR and western blotting in BC cells when *circRPAP2* was depleted or overexpressed. **C** and **D** Expression levels of PTK2 mRNA and protein were detected by qRT-PCR and western blotting in BC cells when *SRSF1* was knocked down or overexpressed. **E** Expression level of *PTK2* pre-mRNA was detected by qRT-PCR in BC cells when *circRPAP2* was depleted or overexpressed. **F** Expression level of *PTK2* pre-mRNA were detected by qRT-PCR in BC cells when *SRSF1* was knocked down or overexpressed. **G** *PTK2 mRNA* is upregulated in BC tissues (*n* = 102). **H** *PTK2 mRNA* is upregulated in BC cell lines. **I** *PTK2 pre-mRNA* is downregulated in BC tissues (*n* = 102). **J** *PTK2 pre-mRNA* is downregulated in BC cell lines. **K** Negative correlation between the expression of *circRPAP2* and *PTK2 mRNA* in BC tissue samples. (*n* = 102, Y = −1.503*X + 2.810, *R*^2^ = 0.662, *P* < 0.001). **L** Positive correlation between the expression of *circRPAP2* and *PTK2 pre-mRNA* in BC tissue samples. (*n* = 102, Y = 1.339*X – 3.063, R^2^ = 0.605, *P* < 0.001). (*P < 0.05, **P < 0.01, ***P < 0.001, ****P < 0.0001).

To further assess the effect of *circRPAP2* binding with SRSF1 in BC, we investigated the molecular pathways deregulated by SRSF1. A previous study reported that SRSF1 could regulate alternative splicing in BC and that splicing targets, including *PTK2*, were involved in SRSF1 oncogenic activity [20]. To comprehensively investigate the ability of *circRPAP2* and SRSF1 to regulate the expression of PTK2, qRT-PCR and western blotting were applied to detect the mRNA, pre-mRNA, and protein expression of PTK2 in MCF-7 and MDA-MB-231 cells transfected with *si-circRPAP2*, *LV-circRPAP2*, *si-SRSF1*, or *LV-SRSF1*. The results of these assays indicated that *circRPAP2* depletion increased the expression levels of PTK2 mRNA and protein, whereas these were decreased by *circRPAP2* overexpression (Fig. 4A and B). Consistently, knockdown or overexpression of SRSF1 downregulated or upregulated the mRNA and protein expression of PTK2 (Fig. 4C and D). Furthermore, the level of *PTK2* pre-mRNA was reduced in BC cells when *circRPAP2* was depleted or SRSF1 was overexpressed, whereas *circRPAP2* overexpression or knockdown of *SRSF1* caused intracellular accumulation of *PTK2* pre-mRNA (Fig. 4E and F). We also analyzed the expression levels of PTK2 mRNA and pre-mRNA in 102 pairs of BC and adjacent normal tissues. The results showed that PTK2 mRNA expression was upregulated (90/102, 88.24%) (Fig. 4G), whereas pre-mRNA expression was downregulated (95/102, 93.14%) (Fig. 4I) in BC tissues compared with that in the adjacent normal tissues. Likewise, the expression of PTK2 mRNA was also significantly increased (Fig. 4H), whereas the expression of pre-mRNA decreased (Fig. 4J) in BC cell lines (MCF-7, MDA-MB-231, and BT-549) compared with that in MCF-10 A. What’s more, the expression level of PTK2 mRNA was negatively linearly correlated with that of circRPAP2 (Fig. 4K), whereas the expression level of PTK2 pre-mRNA was positively linearly correlated with that of circRPAP2 (Fig. 4L) in 102 BC tissue samples. Together, these results suggest that *circRPAP2* and SRSF1 are involved in the alternative splicing of *PTK2* in the BC cell nucleus.

**Fig. 5:**
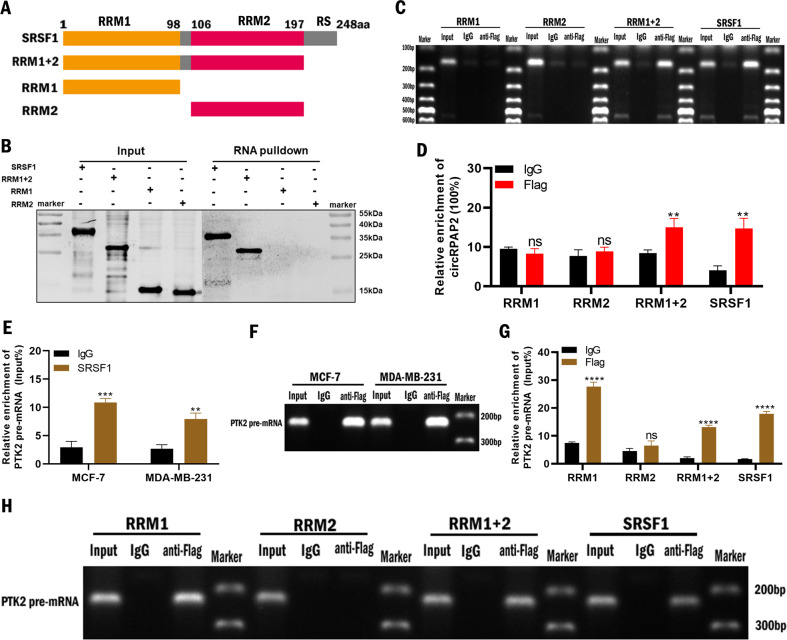
*CircRPAP2* likely competes with the binding between *PTK2* pre-mRNA and RRM1 domain of SRSF1. **A** Design of full-length and truncated *SRSF1* plasmids with FLAG tags. **B–D** RNA pulldown and RIP assays were performed to evaluate the binding between *circRPAP2* and various SRSF1 domains. **E** and **F** RIP assays were performed to explore the binding between SRSF1 and *PTK2* pre-mRNA. **G** and **H** RIP assays were performed to evaluate the binding site between SRSF1 domains and *PTK2* pre-mRNA. (*P < 0.05, **P < 0.01, ***P < 0.001, ****P < 0.0001).

SRSF1 comprises an RS domain and two RNA-recognition motifs (RRM1 and RRM2) responsible for its specific interaction with RNA [21]. To identify the regions of SRSF1 responsible for its binding with *circRPAP2*, we constructed a series of SRSF1 domain mutants (Fig. 5A). RNA pulldown assays and subsequent western blotting were performed in 293 T cells following transfection with the domain mutants. The results exhibited that *circRPAP2* was bound to both RRM1 and RRM2 (Fig. 5B). The specificity of this binding was further substantiated using RIP assays (Fig. 5C and D). Moreover, RIP assays also revealed that SRSF1 could bind to *PTK2* pre-mRNA (Fig. 5E and F), with the subsequent evaluation in 293 T cells following transfection with domain mutants revealing RRM1 as the unique binding site (Fig. 5G and H). Overall, these results suggested that *circRPAP2* likely competed with *PTK2* pre-mRNA to bind with the RRM1 domain of SRSF1, thereby attenuating the alternative splicing of *PTK2*.

**Fig. 6:**
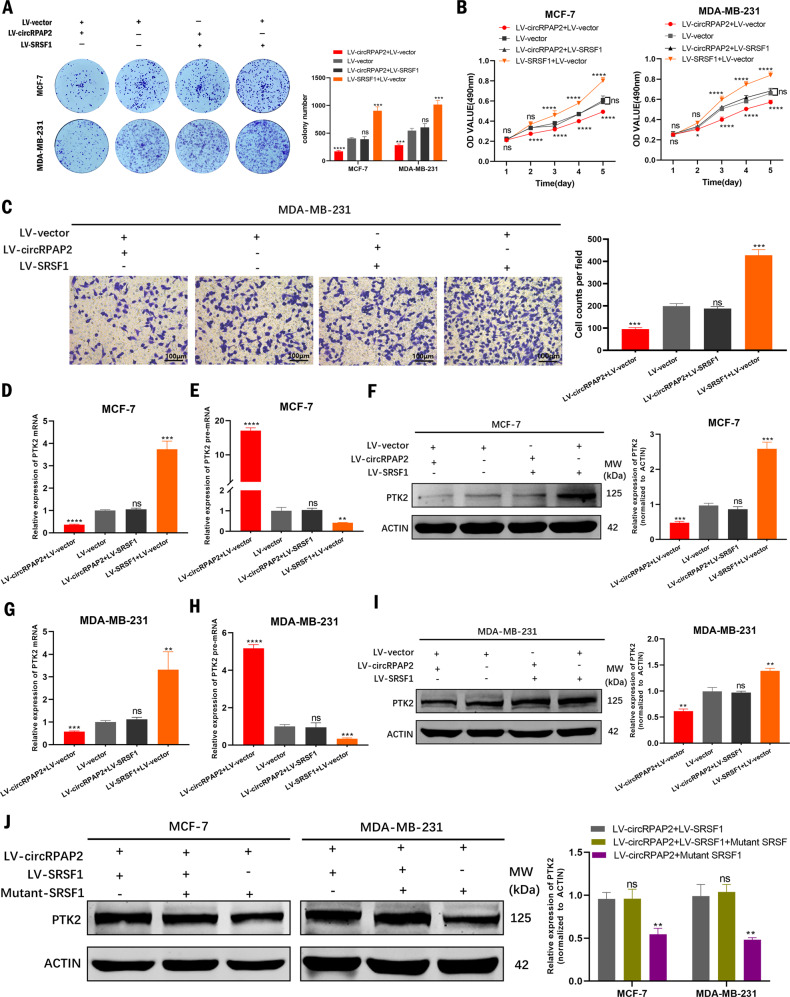
Rescue assay of the *circRPAP2*/SRSF1/PTK2 axis. **A**–**C** *CircRPAP2* overexpression-induced cell proliferation and migration can be rescued by overexpression of SRSF1 as determined using by colony formation (**A**), MTT (**B**), and Transwell migration assays (**C**). **D**–**I** *CircRPAP2* overexpression-mediated reduction in PTK2 mRNA and protein expression and increase in *PTK2* pre-mRNA could be recovered by overexpressing SRSF1 in BC cells as determined by qRT-PCR and western blotting. **J** *CircRPAP2* overexpression-mediated reduction in PTK2 protein expression could not be recovered by mutant of SRSF1 binding site in BC cells as determined by western blotting. (*P < 0.05, **P < 0.01, ***P < 0.001, ****P < 0.0001, ns: no significance).

Accordingly, rescue assays were then conducted to verify that *circRPAP2* regulated the alternative splicing of *PTK2* by binding to SRSF1. We transfected a combination of *LV-circRPAP2* and *LV-SRSF1* to detect the effects on cell proliferative and migratory abilities as well as the expression of *PTK2* in BC cells. The results of colony formation, MTT, and Transwell migration assays indicated that the inhibition of cell proliferation and migration induced by *circRPAP2* overexpression could be rescued by overexpressing SRSF1 (Fig. 6A–C). The results of qRT-PCR demonstrated that *circRPAP2* overexpression reduced *PTK2* mRNA expression and elevated *PTK2* pre-mRNA levels could be recovered by overexpressing SRSF1 in MCF-7 (Fig. 6D and E) and MDA-MB-231 cells (Fig. 6G and H). Western blotting also indicated that *circRPAP2* overexpression decreased the protein expression of PTK2, which could be rescued by the overexpression of SRSF1 (Fig. 6F and I), but not the mutant of SRSF1 binding site (Fig. 6J). These findings validated that *circRPAP2* inhibited BC cell proliferation and migration by binding to SRSF1 to regulate the alternative splicing of *PTK2*.

### *CircRPAP2* inhibits BC growth in vivo

**Fig. 7:**
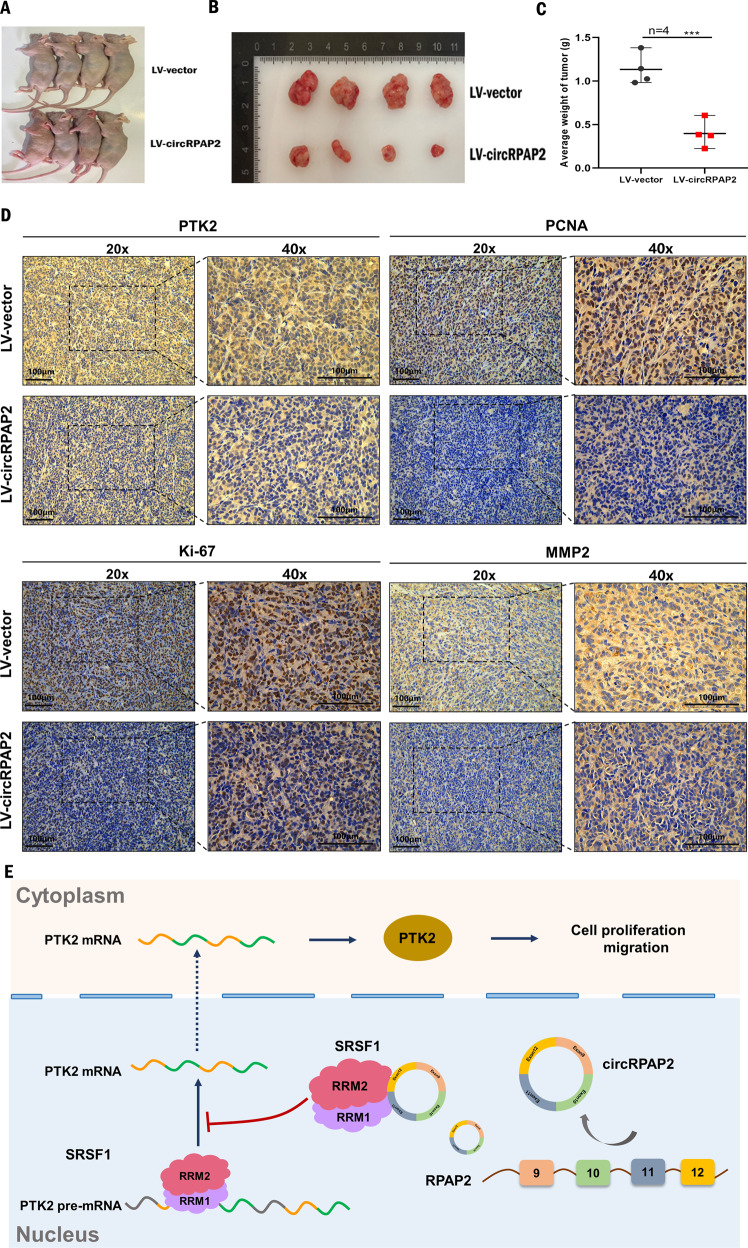
*CircRPAP2* inhibits BC growth in vivo. **A** Representative images of female nude mice injected with MDA-MB-231 cells (4 mice per group). **B** Representative images of xenograft tumors in nude mice. **C** Average tumor weight of nude mice. **D** IHC staining of PTK2, PCNA, Ki-67, and MMP2 in xenograft tumors. (*P < 0.05, **P < 0.01, ***P < 0.001, ****P < 0.0001).

MDA-MB-231 xenograft models were established to confirm the suppressive effect of *circRPAP2* on BC cells in vivo. We first steadily transfected MDA-MB-231 cells with *LV-circRPAP2* or LV-vector and then injected these cells subcutaneously into female nude mice (Fig. 7A). Mouse tumors were photographed, measured, and weighed, verifying that *circRPAP2* overexpression could significantly decrease the diameter and weight of the tumors compared with the negative controls (Fig. 7B and C). Moreover, the xenograft tumors were collected and subjected to immunohistochemistry (IHC) to detect the expression of PTK2, PCNA, Ki-67, and MMP2. As shown in Fig. 7D, the expression of PTK2, PCNA, Ki-67, and MMP2 decreased in *LV-circRPAP2* group compared with that in LV-vector group. Together, the in vivo and in vitro results verified that *circRPAP2* plays a role in suppressing tumors in BC via the *circRPAP2*/SRSF1/PTK2 axis. The proposed mechanism is shown in Fig. 7E.

## Discussion

To date, numerous circRNAs have been identified in the human genome through advances in sequencing technology, and several circRNAs have been confirmed to participate in the genesis of multiple cancers, including BC [22, 23]. *CircRPAP2* was first reported to be differentially expressed in BC plasma compared with that in normal subjects [18], but its function and mechanism have not been reported in BC or other cancers. In this study, we found that *cricRPAP2* was downregulated in BC tissues and cell lines. The underlying cause of *circRPAP2* downregulation in BC may be mediated by EIF4A3, which is a core component of the exon junction complex and plays an essential role in the cyclization of circRNAs [24, 25]. We discovered 3 putative binding sites of EIF4A3 in the upstream and downstream region of the host gene RPAP2 pre-mRNA using Circinteractome (https://circinteractome.nia.nih.gov/). We speculated that EIF4A3 might regulate the splicing of RPAP2 pre-mRNA to inhibit the expression of circRPAP2, and it certainly needs to be confirmed by further experimental results. In vitro gain- and loss-of-function experiments suggested that *circRPAP2* inhibited the proliferation and migration of BC cells, and *circRPAP2* overexpression inhibited BC genesis in vivo. Consistent with this, patients presenting a low expression of *circRPAP2* had a higher rate of axillary lymph node metastasis and later TNM stage than those with high expression of *circRPAP2* among the BC patients included in this study. These results provided evidence that circRPAP2 may constitute a biomarker for BC. However, no significant associations with Ki-67 was observed, and more clinical samples are needed for further investigation.

The functions of circRNAs are related to their subcellular localization [26]. The most common function of cytoplasmic circRNAs is as an miRNA sponge [27], such as circRNA-002178, which is localized to the cytoplasm and acts as a ceRNA to promote PDL1/PD1 expression in lung adenocarcinoma [28]. Nuclear circRNAs have various functions, including interacting with RBPs. For example, binding of the circular RNA cia-cGAS to the DNA sensor cGAS in the nucleus blocks its synthase activity, protecting dormant long-term hematopoietic stem cells from cGAS-mediated exhaustion [9]. Moreover, circRNAs play a vital role in regulating alternative splicing [29]. For instance, a circRNA from *SEPALLATA3* can bind with its host gene DNA locus, forming an RNA:DNA hybrid, to regulate the splicing of host gene mRNA; [30] circ-UBR5 can recruit the splicing factors QKI and NOVA1 and block their splicing functions to change the alternative splicing outcome [31]. This study presents a nuclear circRNA, *circRPAP2* regulates alternative splicing by binding to the splicing factor SRSF1 in BC, and this highlights a new regulatory mechanism for BC mediated by circRNA manipulation.

The oncoprotein SRSF1 [19] has been demonstrated to bind specifically to RNA as shown by the systematic evolution of ligands by exponential enrichment (SELEX) [32], mediated by two RNA recognition motifs (RRM1 and RRM2) [19, 21, 33]. Moreover, SRSF1 has recently been confirmed to bind with non-coding RNAs [34, 35]. Utilizing a series of SRSF1 domain mutants in conjunction with RNA pull-down and RIP assays, we revealed that both RRM1 and RRM2 were required for *circRPAP2* and SRSF1 binding. This result may be related to the circular structure of *circRPAP2*. It is interesting to note that SRSF1 could regulate the biogenesis of circRNA and result in a corresponding change of its host gene linear mRNA level [36]. However, in the present study, SRSF1 did not regulate the expression level of *circRPAP2*, which may be because the host gene of *circRPAP2*, *RPAP2*, was not the splicing target of SRSF1. As the binding of *circRPAP2* and SRSF1 may affect the splicing function of SRSF1, we speculate that the binding of circRPAP2 and SRSF1 would regulate the expression of the circRNAs generated by SRSF1 splicing, but the underlying mechanisms need further investigation.

SRSF1 is involved in the regulation of RNA alternative splicing [20, 37], with the N-terminal extremity of the RRM1 domain playing an important role in pre-mRNA splicing [38]. A previous study reported that *PTK2*, also known as *FAK*, constitutes a splicing target of SRSF1 [20]. *PTK2* encodes a tyrosine kinase that plays a vital role in cellular communication, especially in cell signaling systems [39, 40]. Moreover, the mRNA and protein levels, and activation of PTK2 are positively associated with cell proliferation, migration, and survival [40–42]. In the present study, the alternative splicing of *PTK2* was found to be regulated by *circRPAP2* and SRSF1, with *PTK2* pre-mRNA able to bind with the RRM1 domain of SRSF1. However, the identification that RRM1 also binds to *circRPAP2* suggests that competition is likely between *circRPAP2* and *PTK2* pre-mRNA. When *circRPAP2* binds to SRSF1, SRSF1 does not appear to bind to *PTK2* pre-mRNA, interfering with the alternative splicing effect of SRSF1 on *PTK2* and leading to the reduction in PTK2 mRNA and protein expression, ultimately inhibiting the proliferation and migration of BC.

In conclusion, this study identified a nuclear circRNA, *circRPAP2*, that is downregulated in BC tissues and cell lines. *CircRPAP2* plays a tumor suppressor role in BC in vitro and in vivo. Specifically, *circRPAP2* likely disrupts the alternative splicing function of SRSF1 on *PTK2* pre-mRNA by binding with SRSF1 RNA recognition motifs. Together, our findings highlight *circRPAP2* as a potential biomarker for BC and reveal the *circRPAP2*/SRSF1/PTK2 axis as a new regulatory mechanism for BC.

## Materials and methods

### Patients and samples

The BC inclusion criteria were as follows: (1) patients with a diagnosis of BC confirmed by the pathological report; (2) patients without any preoperative radiotherapy or chemotherapy; and (3) patients without any other malignancies or severe chronic disease. Patients with BC who underwent surgery at Shanghai Tenth People’s Hospital of Tongji University and met the above criteria between 2017 and 2020 were enrolled in this study. BC tissues and their adjacent normal tissues were immediately snap-frozen in liquid nitrogen until use. This study was approved by the institutional Ethics Committee of Shanghai Tenth People’s Hospital (No.2020-KN174-01). All participants provided their consent to the investigators for participation in the study, and the study methodology adhered to standards outlined in the Declaration of Helsinki.

### RNA isolation, qRT-PCR, PCR, and agarose gel electrophoresis assay

The total RNA from paired BC tissues or cells was extracted using TRIzol^®^ reagent (Invitrogen, Carlsbad, CA, USA) according to the manufacturer’s protocol. cDNA was synthesized using the HiScript III RT SuperMix kit (Vazyme Biotech, Nanjing, China). qRT-PCR was conducted using the SYBR Green Master Mix (Yeasen, Shanghai, China). Primer sequences were designed and synthesized by RiboBio (Guangzhou, China). 18 S was used as an internal reference for circRNAs, and *ACTB* was used as an internal reference for genes. The relative expression of circRNAs was assessed using the threshold cycle (CT) values. The PCR assay was conducted according to manufacturer’s instructions using the 2×Hieff™ PCR Master Mix (with dye) (Yeasen). The PCR products were obtained using different primers were subjected to 2% agarose gel electrophoresis, and the gel was scanned using the Gel Doc XR + imager (Bio-Rad, Hercules, CA, USA).

### Actinomycin D assay

Actinomycin D assays were used to determine RNA stability. When the cell density reached 80%, actinomycin D was added at 0, 8, and 16 h, respectively, to achieve a concentration of 2 μg/mL in the medium, and the cells were collected at 24 h. Subsequently, cell precipitates were obtained after treatment with actinomycin D for 0, 8, 16, and 24 h. The total RNA was extracted from the obtained cells using cell RNA extraction procedure and evaluated using qRT-PCR.

### Cell culture, plasmids, siRNA transfection, and lentiviral transduction

Normal breast epithelial cell line (MCF-10A) and BC cell lines (MCF-7, MDA-MB-231, and BT549) were acquired from the Chinese Academy of Sciences (Shanghai, China). All cells were cultured according to manufacturer’s instructions. The siRNAs targeting *circRPAP2* and *SRSF1* were designed and synthesized by RiboBio. The plasmid *pLV-circRPAP2-Hygro* was designed and synthesized by Haro Life (Shanghai, China) and plasmids encoding *FLAG*-tagged full-length and truncated SRSF1 were designed and synthesized by IBSBio (Shanghai, China). siRNAs and plasmids were transfected into cells using Lipo8000TM Transfection Reagent (Beyotime Jiangsu, China) according to the manufacturer’s instructions. The stable 231-MDA-MB cell line of *LV-circRPAP2* was obtained by selection with hygromycin B (Beyotime).

### Cytoplasm–nucleus separation assay

The Thermo Invitrogen™ PARIS™ kit was used for the cytoplasm–nucleus separation assays to investigate the cellular distribution of *circRPAP2*. The cytoplasm–nucleus separation assay was performed following the manufacturer’s protocol. The expression levels of the cytoplasmic control *GAPDH*, nuclear control U6, and *circRPAP2* were examined by qRT-PCR in the cytoplasmic and nuclear fractions of MCF-7 and MDA-MB-231 cells.

### RNA FISH

The Ribo™ Fluorescent in Situ Hybridization Kit (Ribo) was used in the FISH assay following the manufacturer’s instructions. Specific probes for *circRPAP2* were designed and synthesized by GenePharma (Shanghai, China). Cell Navigator F-Actin Labeling Kit was from AAT Bioquest (CA, USA). A The nuclei were stained with 4,6-diamidino-2-phenylindole (DAPI). A fluorescence microscope (Leica Microsystems, Mannheim, Germany) was used to capture images of the cells.

### MTT and colony formation assays

MTT and colony formation assays were used to assess the proliferation ability of BC cells. The cells at a density of 2,000 cells per well were placed in 96-well plates following treatment. The cells were evaluated using an MTT assay kit (Sigma, St. Louis, MO, USA) according to the manufacturer’s instructions. The cells were assayed at 24, 48, 72, and 96 h using a microplate reader to measure the optical density at 490 nm. For the colony formation assay, the cells at a density of 1,000 cells per well were transferred into six-well plates following treatment. When the colonies were observable, they were washed twice with cold phosphate-buffered saline, fixed with 75% ethanol, and stained with 0.1% crystalline purple. The colonies were photographed and counted.

### Wound healing and Transwell migration assays

MDA-MB-231 cells were transfected with the reagent as indicated in the 6-well plates. When the treated cells reached approximately 90% confluence, a wound was introduced by generating a scratch using a 200 μL pipette tip in each well. The monolayers were washed twice with 1× phosphate-buffered saline and cultured with Dulbecco’s modified Eagle’s medium without fetal bovine serum, excluding the effect of cell proliferation on the results. Wound healing was observed under a light microscope (Leica Microsystems) and photographed at 0 and 36 h at the same position to observe cell migration.

Transwell chambers (Corning, Inc., Lowell, MA, USA) were used to measure the migration ability of MDA-MB-231 cells in 24-well plates. The cells at a density of 3,000 cells were transferred into the upper chamber with 200 µL serum-free medium, and the lower chamber filled with 500 µL medium containing 10% fetal bovine serum. After 24 h, the cells on the opposite side of the filter were fixed with 75% ethanol for 30 min and stained with crystal violet solution for 10 min after removing the cells in the upper chamber. Images were obtained using a microscope (Leica Microsystems), and the migrated cells were counted in five random fields.

### RNA pulldown, silver staining, and mass spectrometry analysis

A probe targeting *circRPAP2* and the anti-sense for RNA pulldown were synthesized by GenePharma, and RNA pulldown was performed using the BersinBio™ RNA pulldown Kit (BersinBio, Guangzhou, China) according to the manufacturer’s protocol. MCF-7 and MDA-MB-231 cells were used for the endogenous RNA pulldown assay, whereas 293 T cells were transfected with FLAG-tagged full-length SRSF1 or SRSF1 domain plasmids for the exogenous RNA pulldown assay. Western blotting was performed using anti-SRSF1 (Santa Cruz Biotechnology, Dallas, TX, USA) and anti-FLAG (Abclonal, Wuhan, China) antibodies. Silver staining was carried out using the Protein Silver Stain Kit (Yeasen) following the manufacturer’s protocol. The mass spectrometry analysis of differential protein bands was conducted by IBSBio.

### Western blotting, RIP, IF, and IHC

Proteins in cells were extracted using RIPA lysis buffer (Beyotime), and concentrations were determined using a protein assay kit (Beyotime). Protein bands were scanned using the Odyssey Infrared scanning system (Li-Cor, Lincoln, NE, USA). The following antibodies were used: anti-PCNA, anti-PTK2 (Abclonal), anti-SRSF1, anti-MMP2, and anti-MMP9 (Santa Cruz Biotechnology). RIP assays were performed using the BersinBio^TM^ RNA Immunoprecipitation (RIP) Kit (BersinBio), with anti-SRSF1 (Santa Cruz Biotechnology), anti-FLAG (Abclonal), and appropriate control IgG (BersinBio) antibodies. Subsequently, qRT-PCR and agarose gel electrophoresis assays were performed. IHC was performed using antibodies against PCNA, Ki-67, PTK2, and MMP2 (Abclonal). IF was performed using an antibody against *SRSF1* (Santa Cruz Biotechnology). Images were taken using a microscope (Leica Microsystems). All assays were performed according to the manufacturer’s protocol. The catalog numbers for all antibodies used were provided in Table S2.

### Xenograft tumor assays

Athymic female nude mice (5 weeks old, 18–22 g) were obtained from Vital River (Beijing, China). Approximately 1 × 10^6^ MDA-MB-231 cells with stable expression of *LV-circRPAP2* or LV-vector were injected into the second mammary fat of the mice (*n* = 4 in each group). Tumor size was measured weekly. After 5 weeks, the mice were euthanized by cervical dislocation. Tumors in each group were collected and weighed. All animal experiments were approved by the Animal Care and Use Committee of the Shanghai Tenth People’s Hospital (No. SHDSYY-2020-0600) and performed in accordance with the guidelines of the Ethics Committee of Tongji University.

### Statistical analysis

Comparisons between paired specimens were analyzed using the Wilcoxon matched-pairs signed rank test, whereas the unpaired Student’s t-test was used for unpaired samples. Two-way analysis of variance (ANOVA) was used to analyze the MTT assay results. In addition, comparisons between circRPAP2 expression levels and patient clinical features were conducted using the chi-square test. Statistical analysis was performed using SPSS (version 25.0, SPSS Inc., Chicago, IL, USA) and GraphPad Prism 8.0 (GraphPad, LaJolla, CA, USA). Data were obtained from three independent experiments and are presented as the mean ± standard deviation (SD). The data were considered statistically significant at *P* < 0.05.

## Supplementary information

Table S1

Table S2

Figure S1

Figure S2

Figure legends for supplemental figures

Original Data File

## Data Availability

All data and materials are available upon request by contacting the corresponding author. The sequence information including all primers, siRNAs, si- NC, and probes in this study can be found in Table S1
