# Supplementary material for: CircRPAP2 regulates the alternative splicing of PTK2 by binding to SRSF1 in breast cancer
Source: Cell Death Discov. 2022 Apr 2;8:152. doi: 10.1038/s41420-022-00965-y (PMC8976847; doi:10.1038/s41420-022-00965-y)
Supplement: Supplementary file 1 — Table S1 [file 41420_2022_965_MOESM1_ESM.docx]

| **Table S1. Primers, siRNAs and probes sequences** | |
| --- | --- |
| Names | Sequences (5'-3') |
| *circRPAP2* |  |
| Forward | ATTCAGAAACATTCTCAGGAAG |
| Reverse | AGTCTATCAGTGGAAAGGTGG |
| 18S |  |
| Forward | TAGAGGGACAAGTGGCGTTC |
| Reverse | CGCTGAGCCAGTCAGTGT |
| RPAP2 |  |
| Forward | AGGGAGTTTTACAGAGGACGG |
| Reverse | ACGATGCGTTTTCTAATCTGGT |
| SRSF1 |  |
| Forward | ATTCCTGCCCCAACCAAACC |
| Reverse | TGCTCCAGCGTTTATCTCCA |
| PTK2 mRNA |  |
| Forward | TGGGCGGAAAGAAATCCTGC |
| Reverse | GGCTTGACACCCTCGTTGTA |
| PTK2 pre-mRNA |  |
| Forward | CTCGGAATGTTCTGGTGTCCT |
| Reverse | GCTGTCGCTTCCTCCATTTAGT |
| GAPDH |  |
| Forward | ACCTCAACTACATGGCTGAGAA |
| Reverse | CCAGTGAGCTTCCCGTTCAG |
| ACTIN |  |
| Forward | CAGAGCCTCGCCTTTGCC |
| Reverse | GTCGCCCACATAGGAATC |
| *circRPAP2* siRNA |  |
| si-circRPAP2 Target sequence | TGTTTACCAGAGTGCATGA |
| si2-circRPAP2 Target sequence | TTACCAGAGTGCATGATTC |
| si3-circRPAP2 Target sequence | GAGTGCATGATTCCACCTT |
| SRSF1 siRNA |  |
| Sense | GCUGAUGUUUACCGAGAUGTT |
| Antisense | CAUCUCGGUAAACAUCAGCTT |
| si-NC |  |
| Sense | UUCUCCGAACGUGUCACGUTT |
| Antisense | ACGUGACACGUUCGGAGAATT |
| *circRPAP2* FISH probe | GTGGAA+TCATGCAC+TCTGGTAAACA |
| *circRPAP2* RNA pulldown probe | GUGGAAUCAUGCACUCUGGUAAACA |
|  |  |
