# Supplementary material for: CircRPAP2 regulates the alternative splicing of PTK2 by binding to SRSF1 in breast cancer
Source: Cell Death Discov. 2022 Apr 2;8:152. doi: 10.1038/s41420-022-00965-y (PMC8976847; doi:10.1038/s41420-022-00965-y)
Supplement: Supplementary file 2 — Table S2 [file 41420_2022_965_MOESM2_ESM.docx]

**Table S2. The catalog numbers for all antibodies**

| **Antibodies** | **Manufacturer** | **Catalog numbers** |
| --- | --- | --- |
| FLAG | Abclonal | AE005 |
| Ki-67 | Abclonal | A16919 |
| PCNA | Abclonal | A0264 |
| PTK2 | Abclonal | A11195 |
| MMP2 | Santa Cruz Biotechnology | sc-13594 |
| MMP9 | Santa Cruz Biotechnology | sc-393859 |
| SRSF1 | Santa Cruz Biotechnology | sc-33652 |
