# Supplementary figures and images for: CircRPAP2 regulates the alternative splicing of PTK2 by binding to SRSF1 in breast cancer
Source: Cell Death Discov. 2022 Apr 2;8:152. doi: 10.1038/s41420-022-00965-y (PMC8976847; doi:10.1038/s41420-022-00965-y)

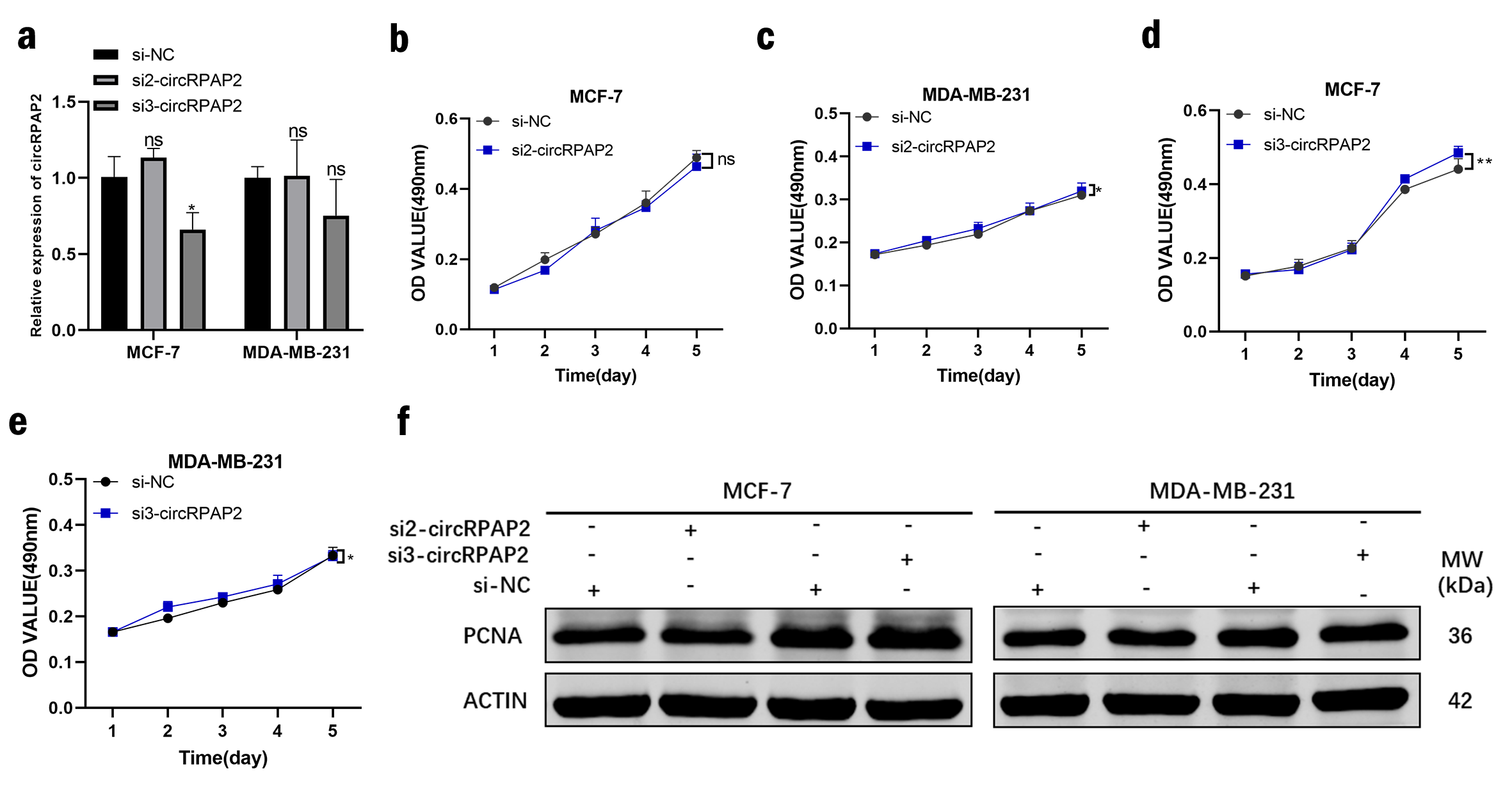

Supplement: Supplementary file 3 — Figure S1 [file 41420_2022_965_MOESM3_ESM.tif]

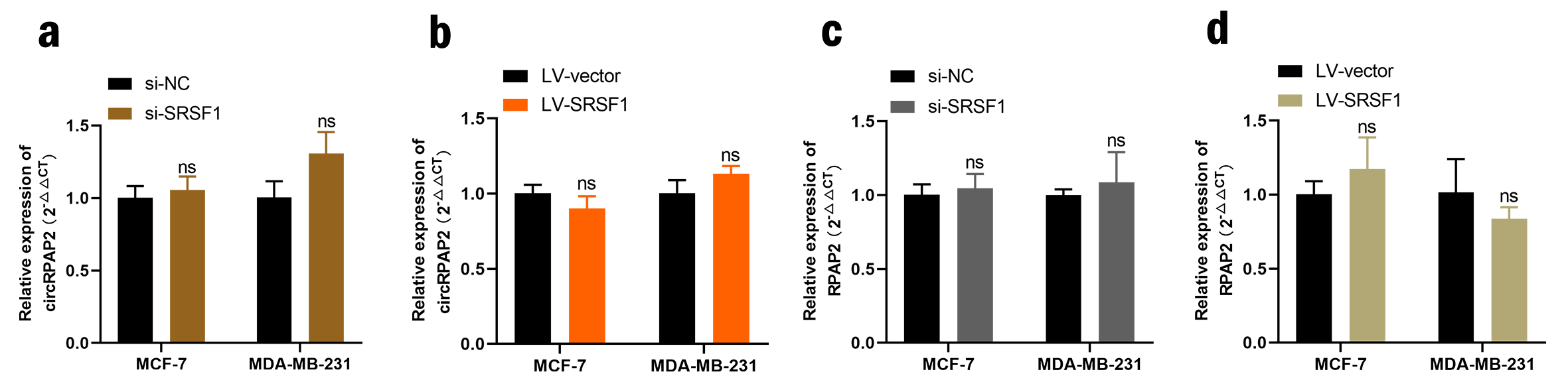

Supplement: Supplementary file 4 — Figure S2 [file 41420_2022_965_MOESM4_ESM.tif]

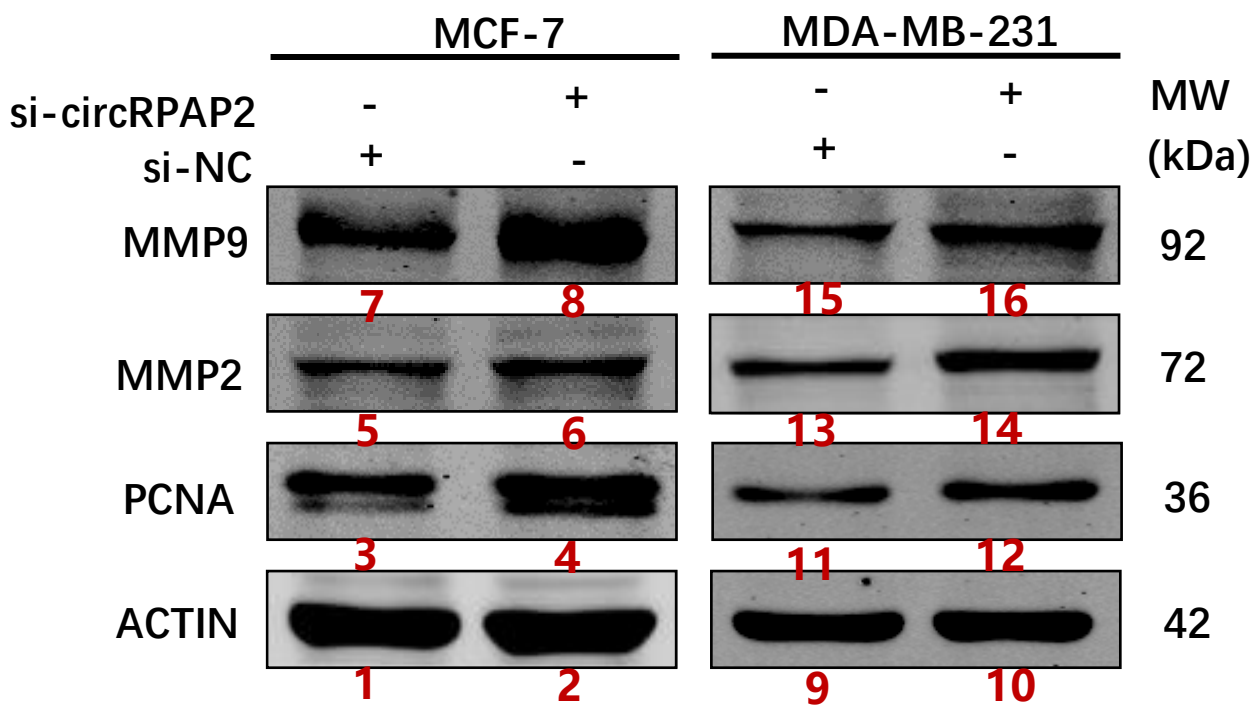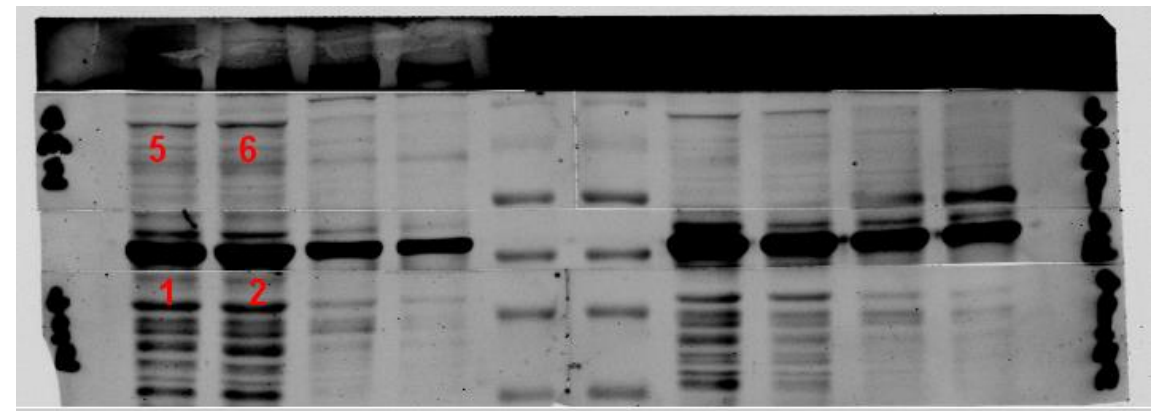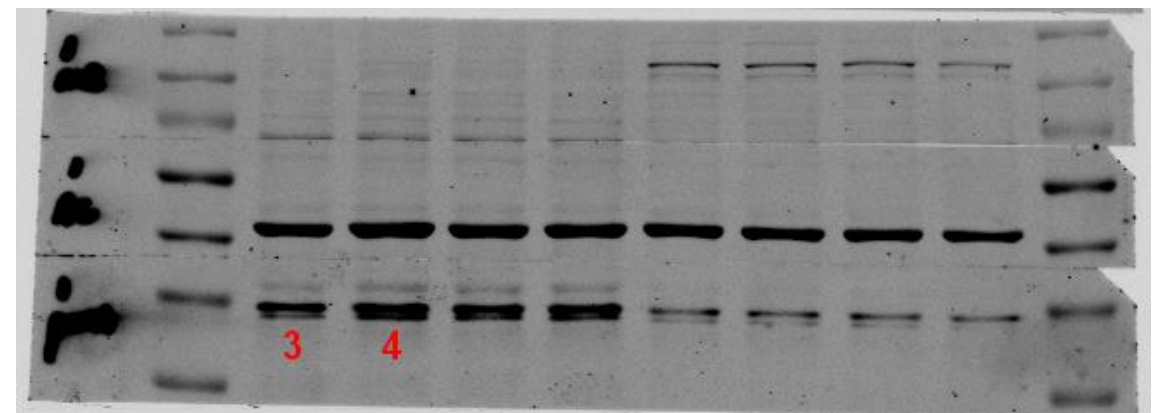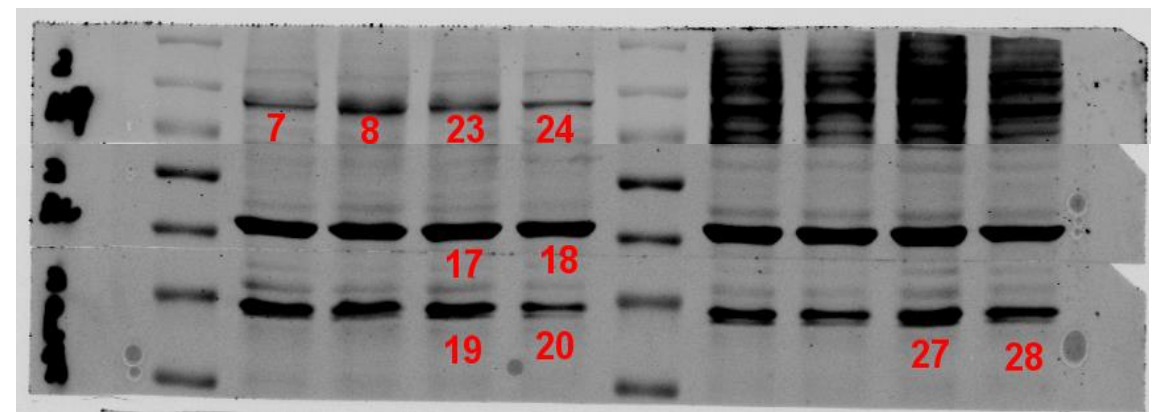

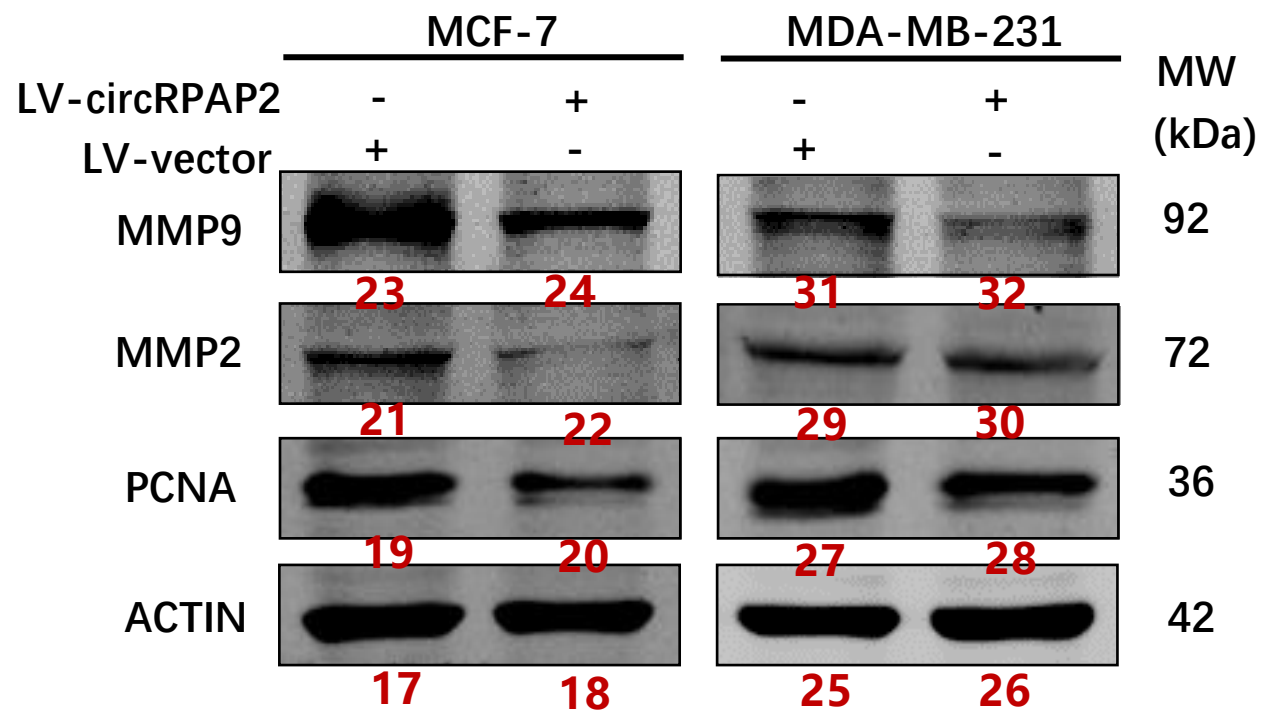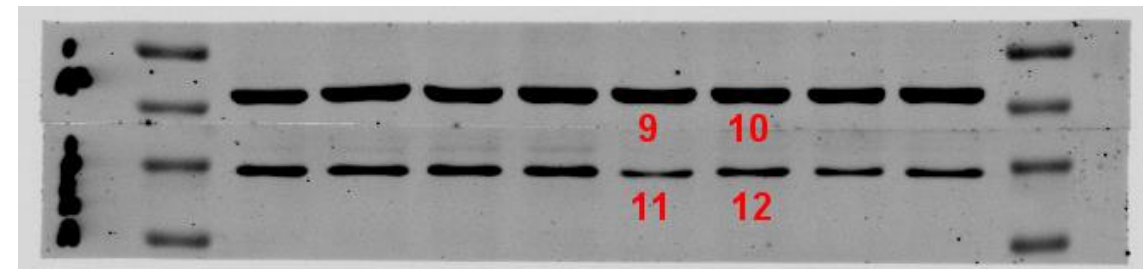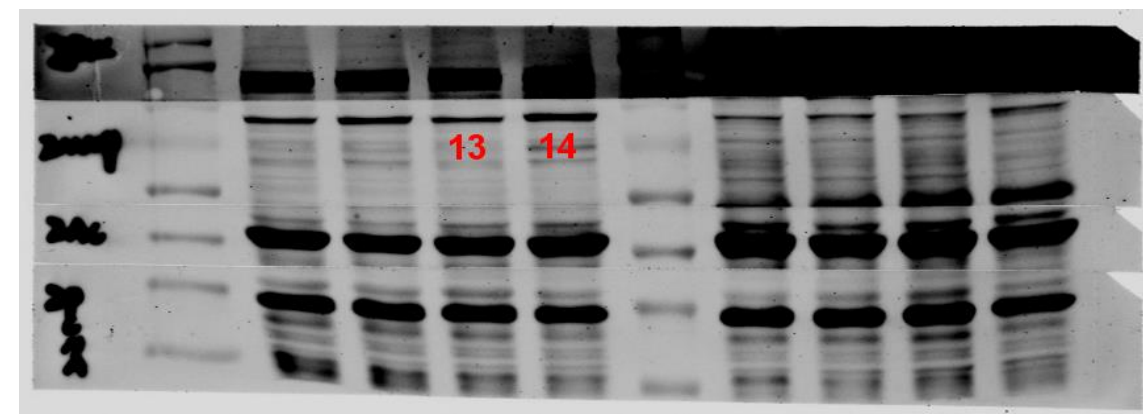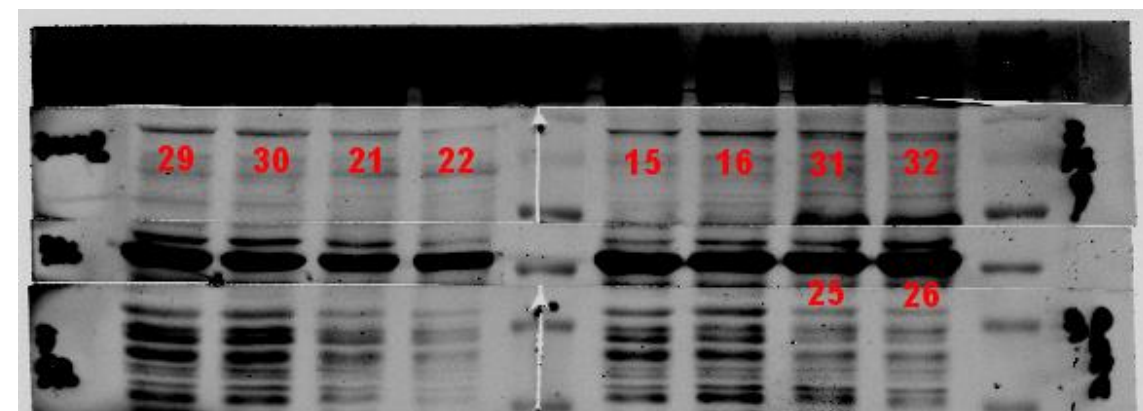

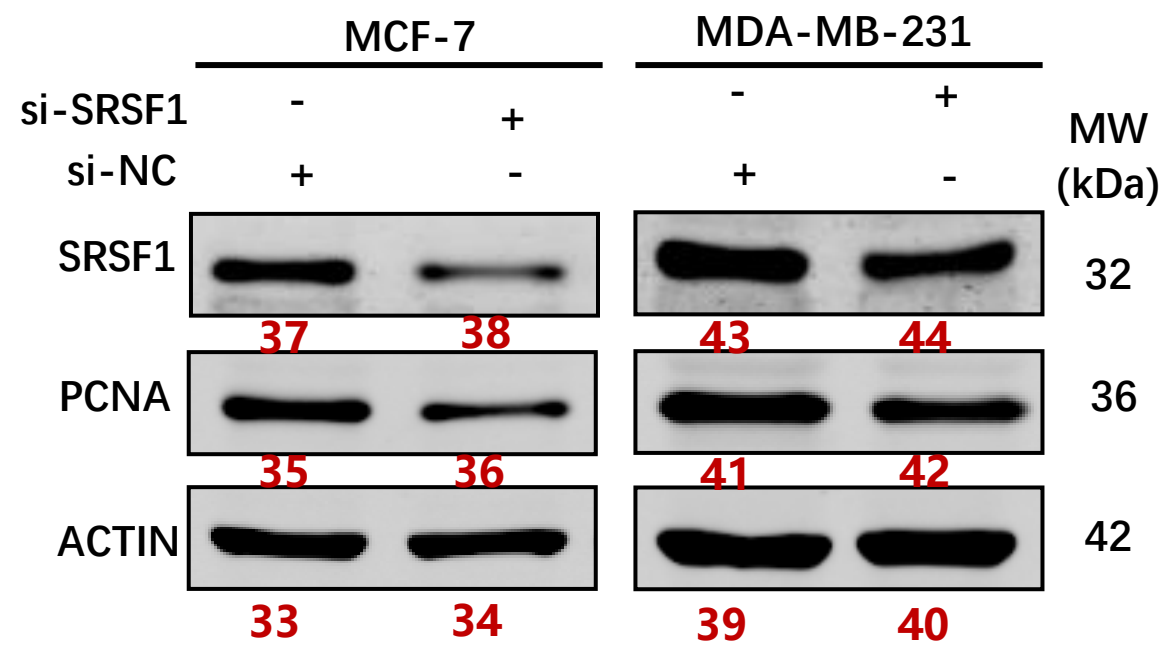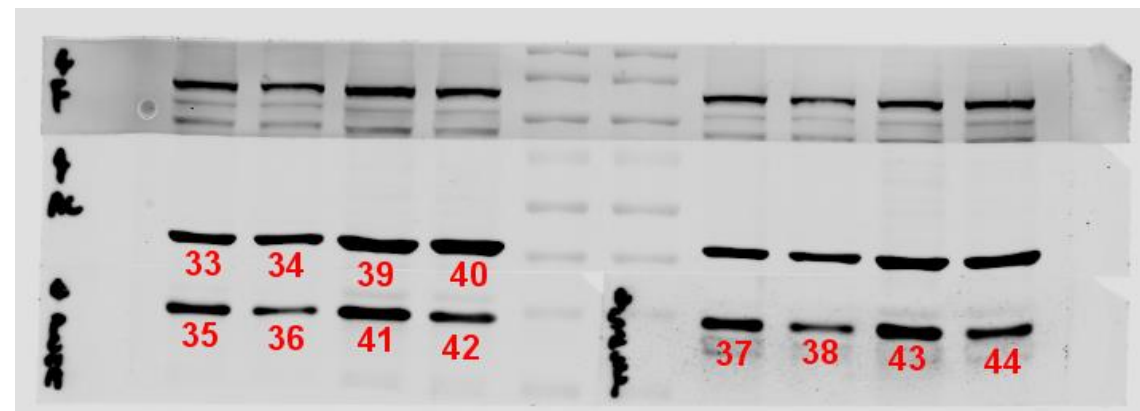

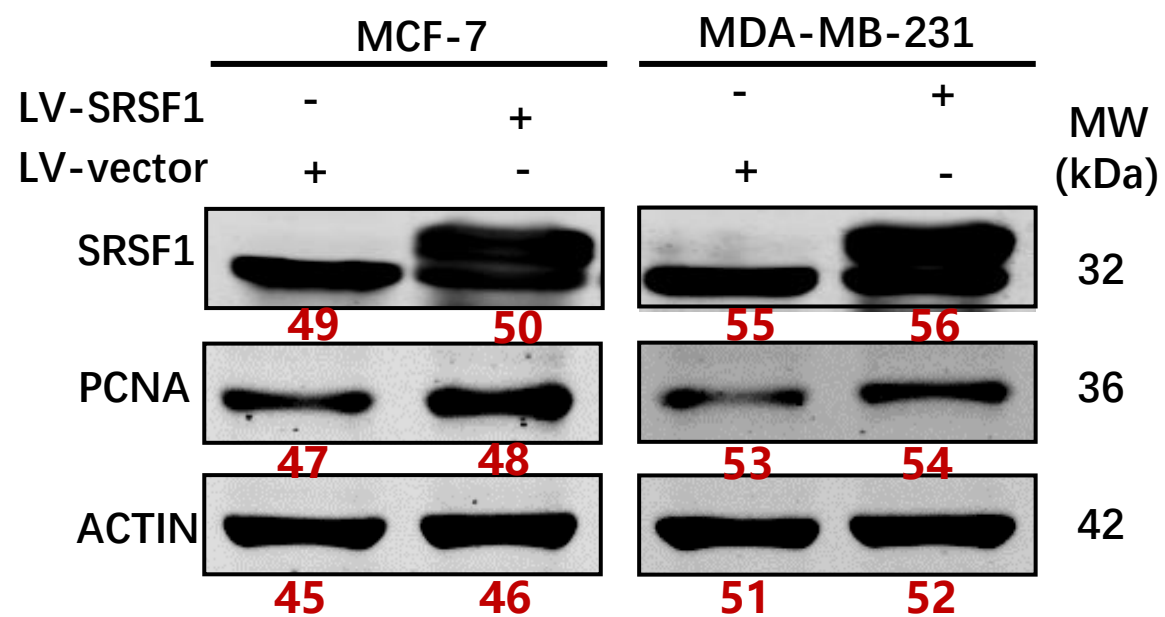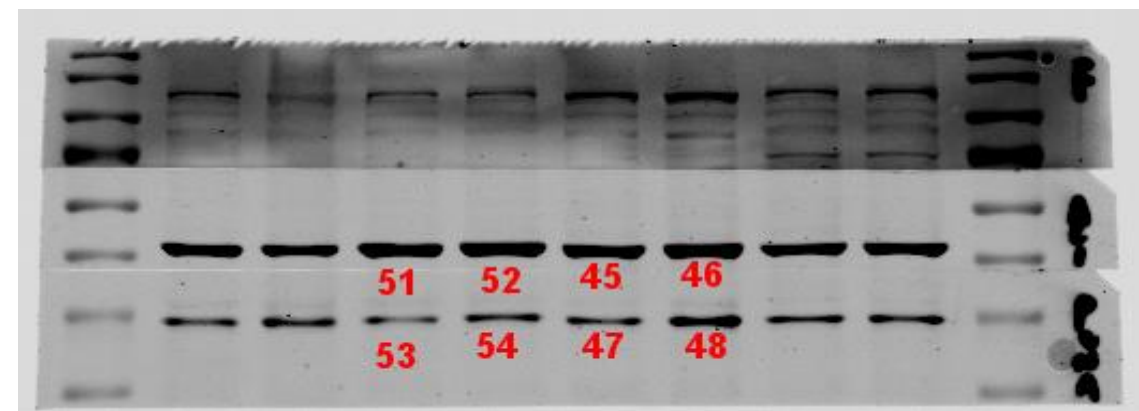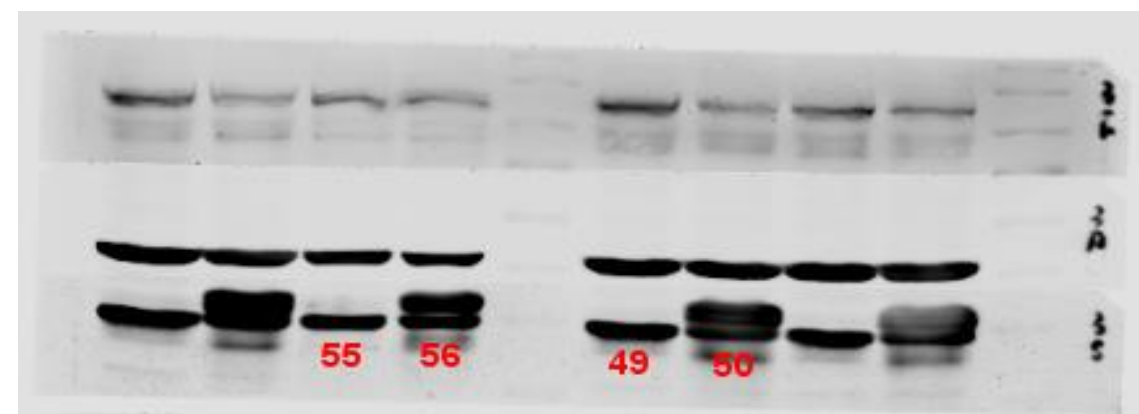

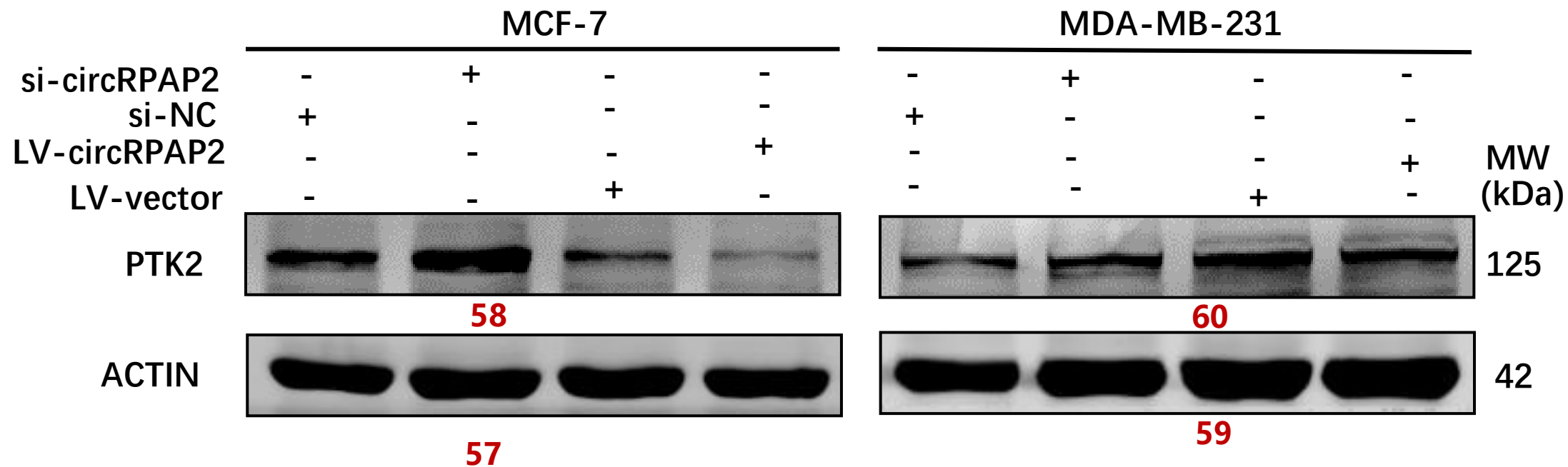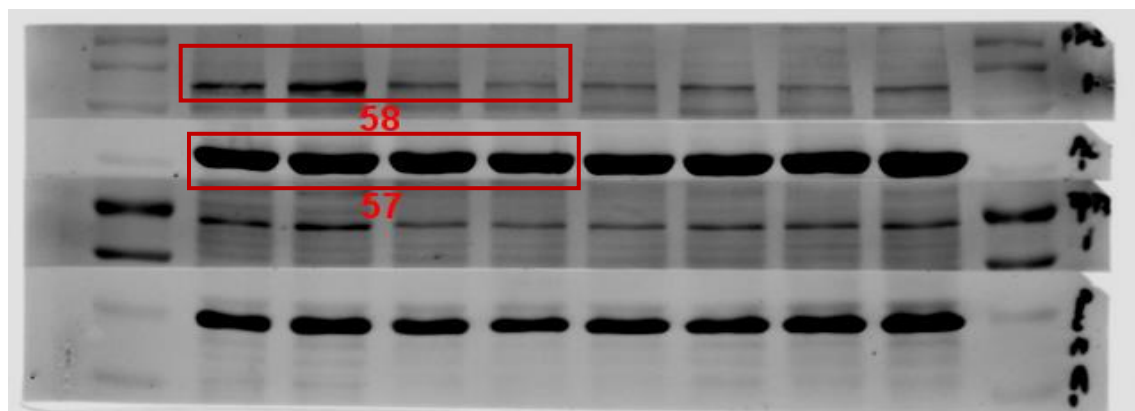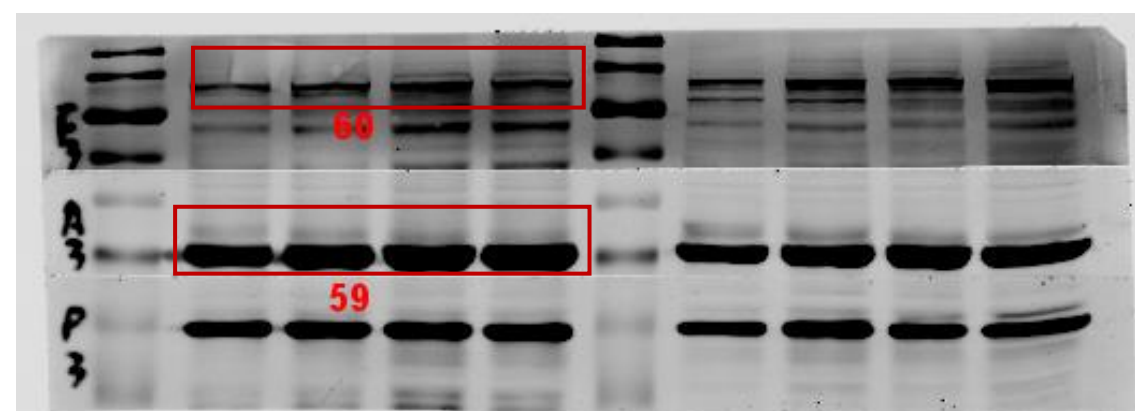

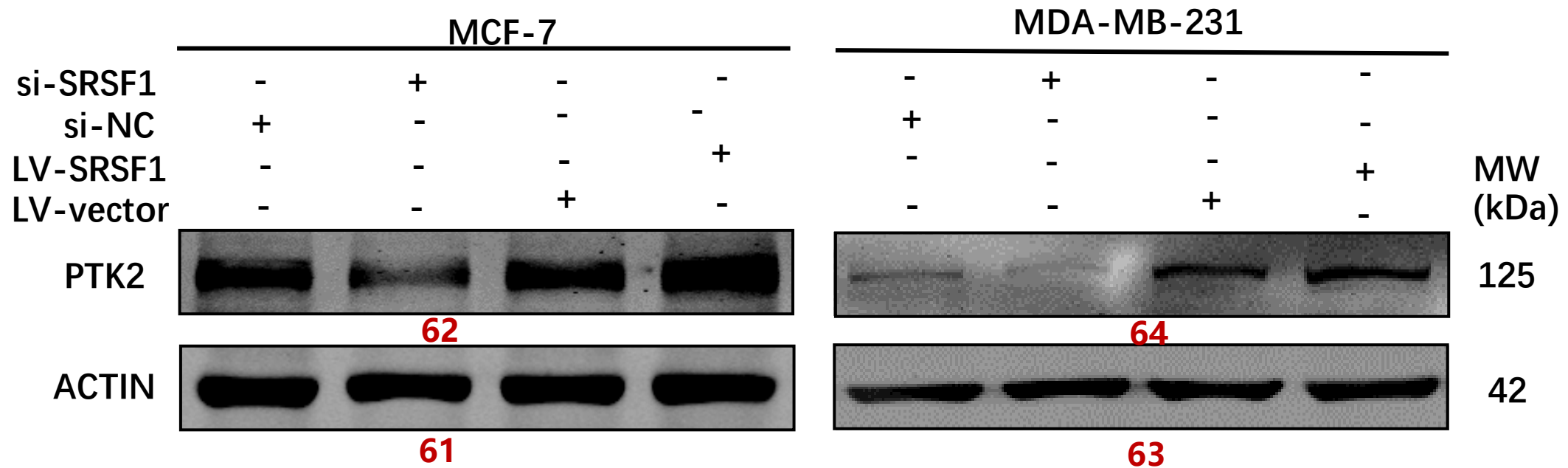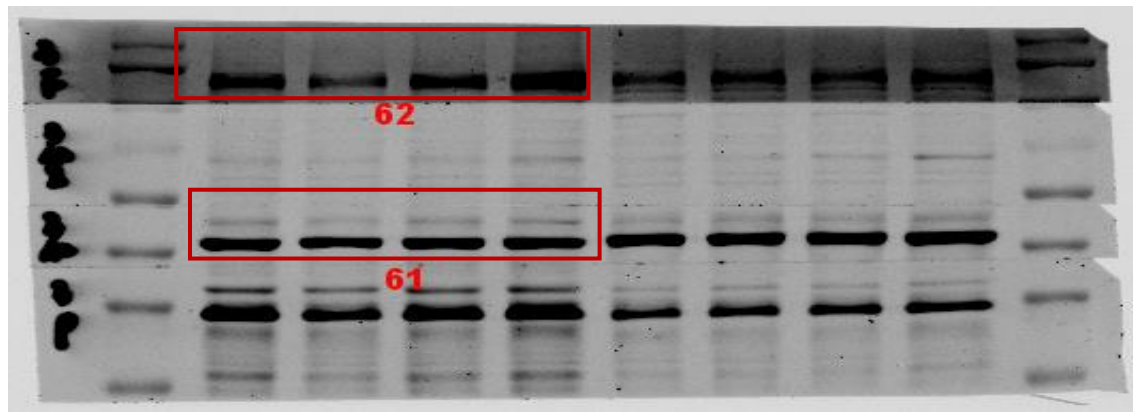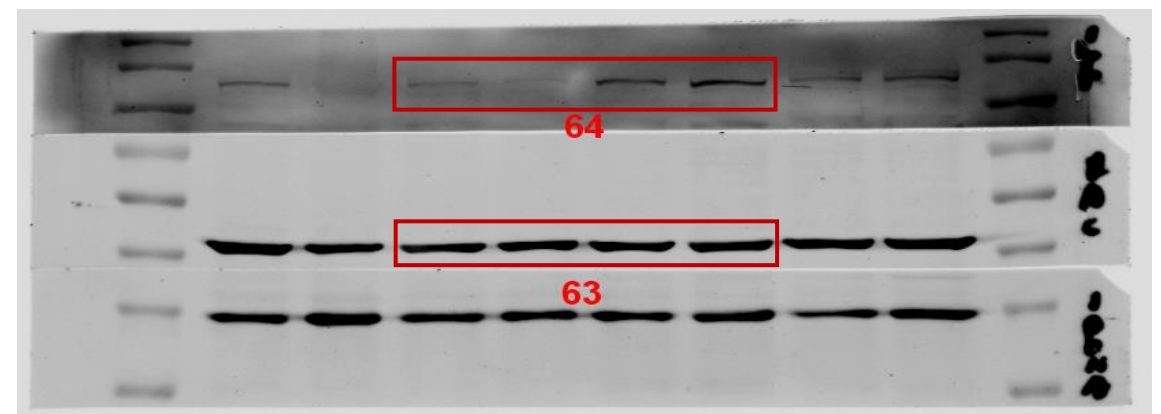

# MCF-7

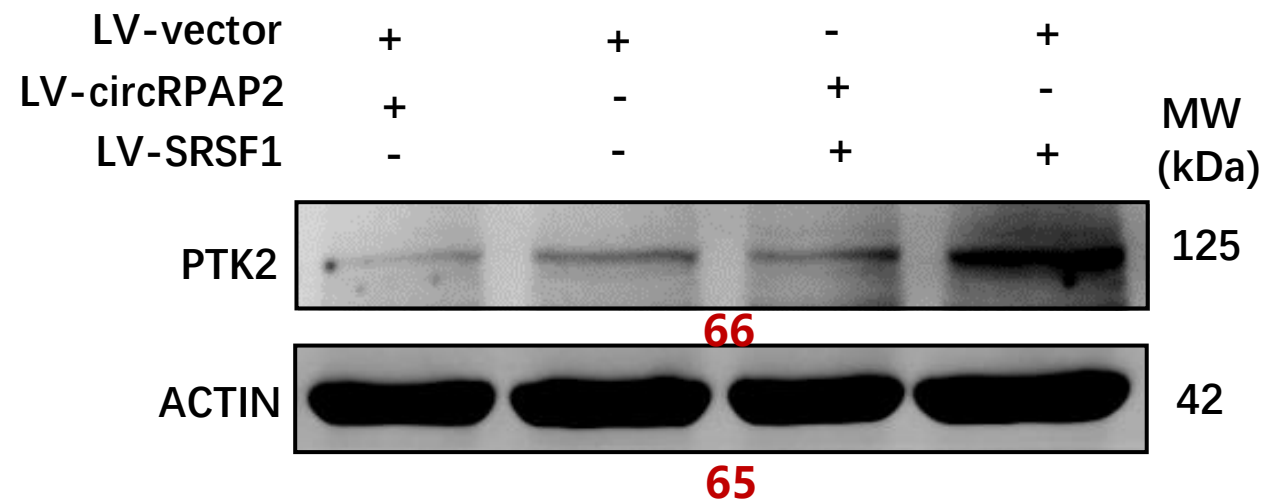

# MDA-MB-231

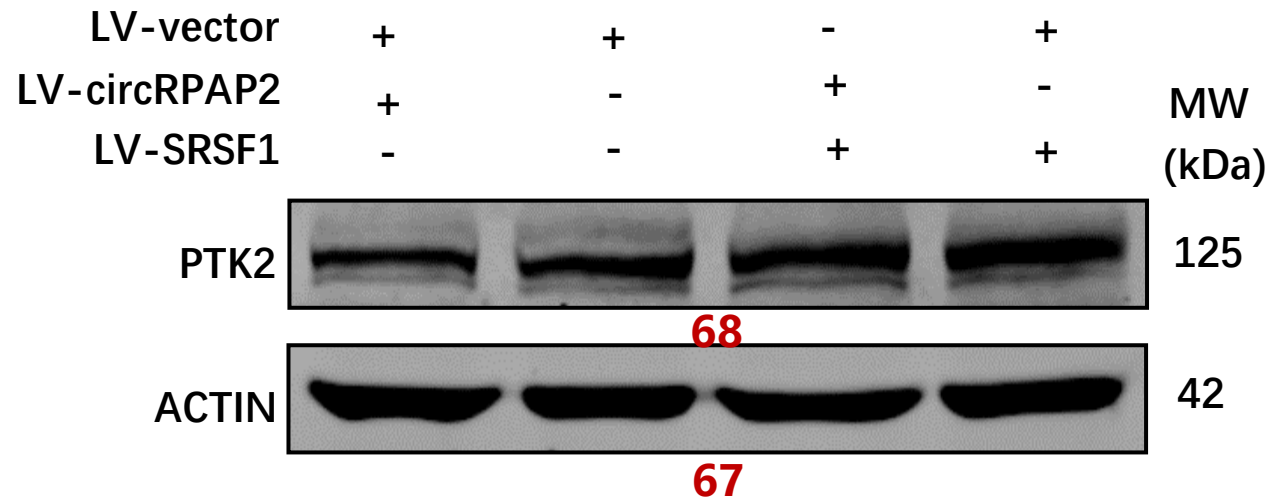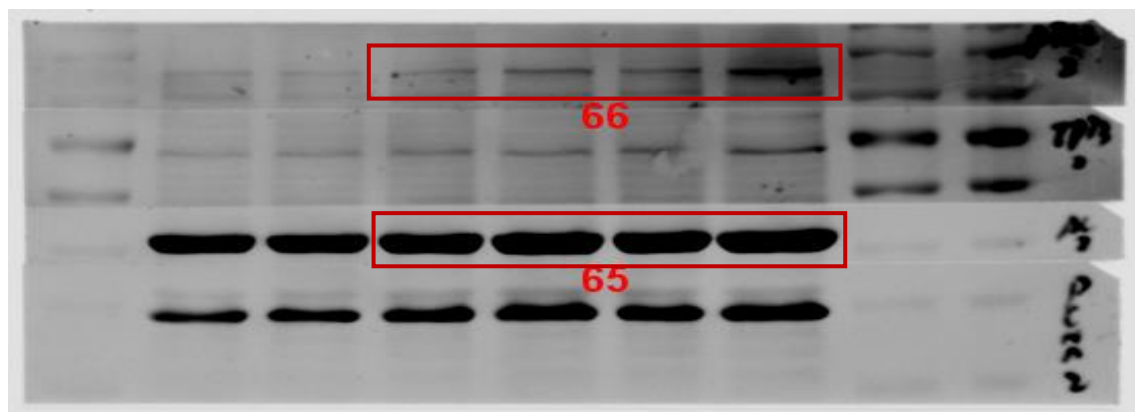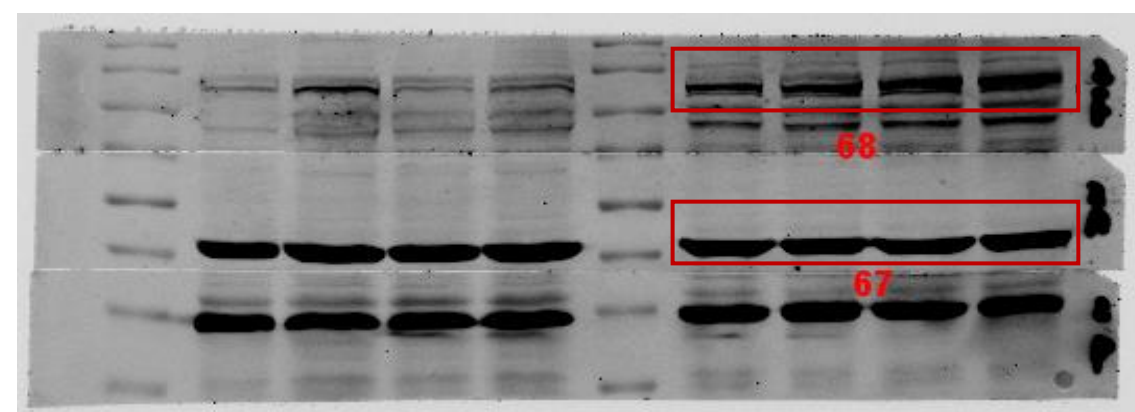

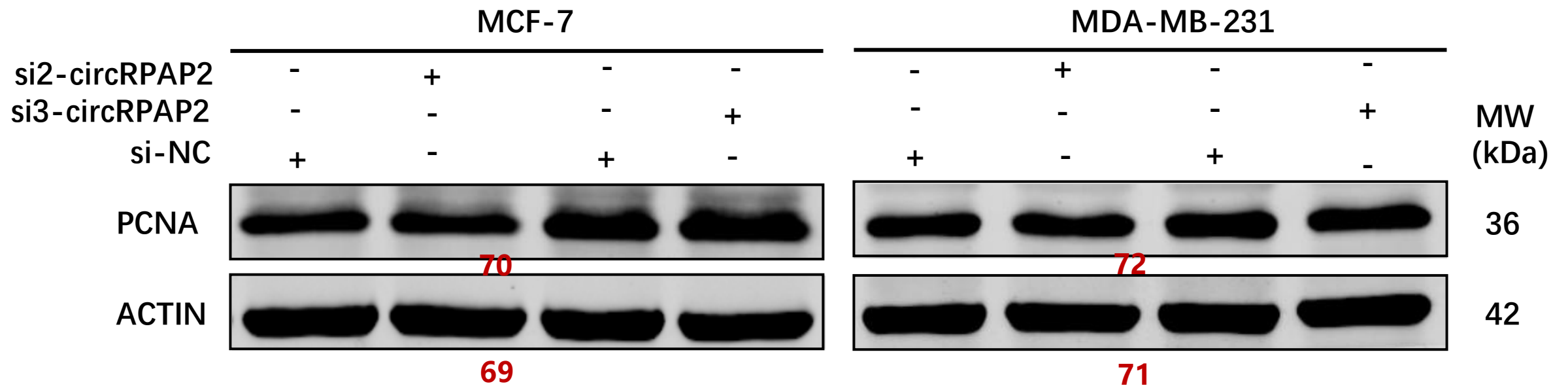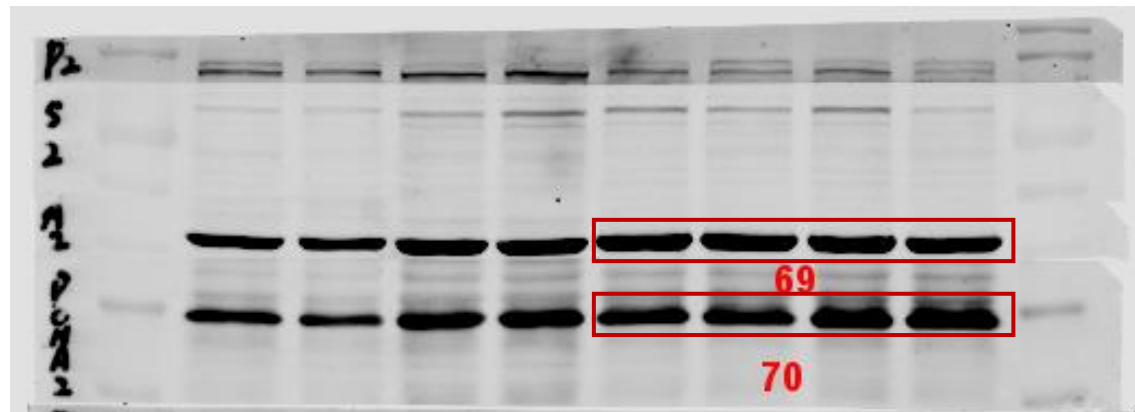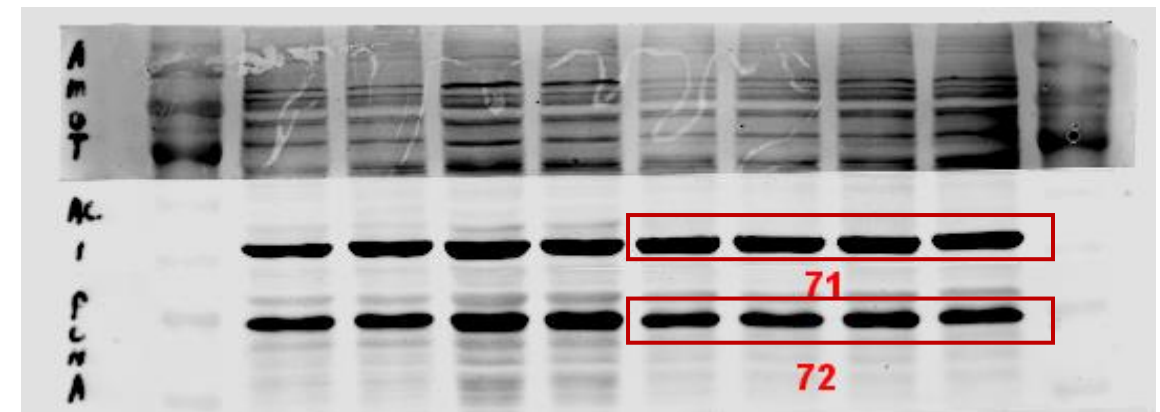

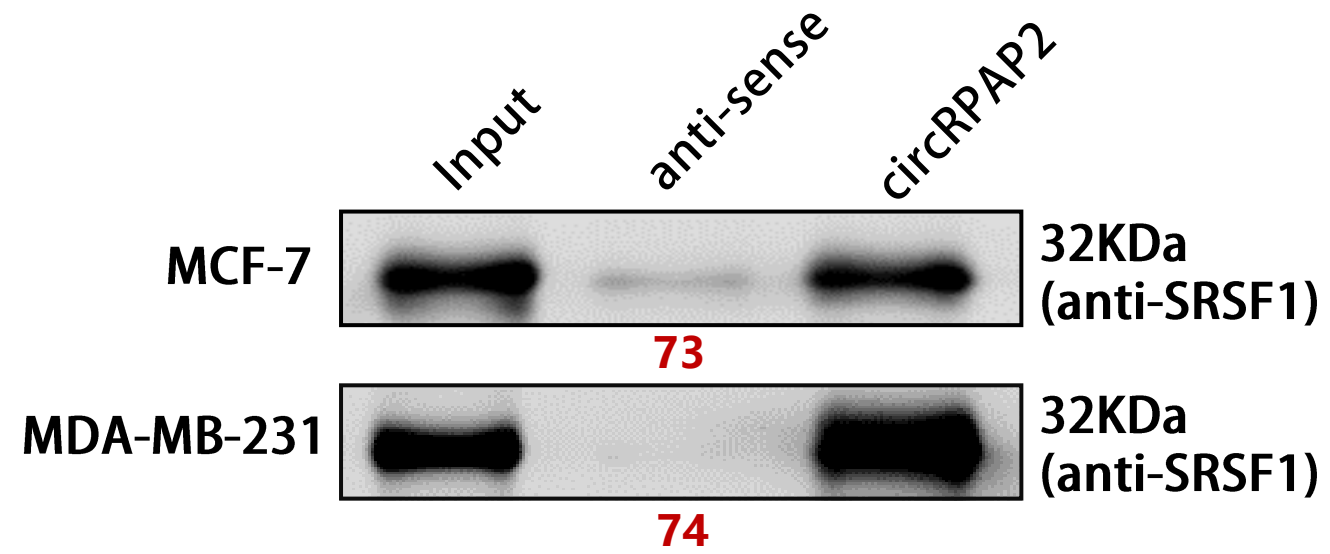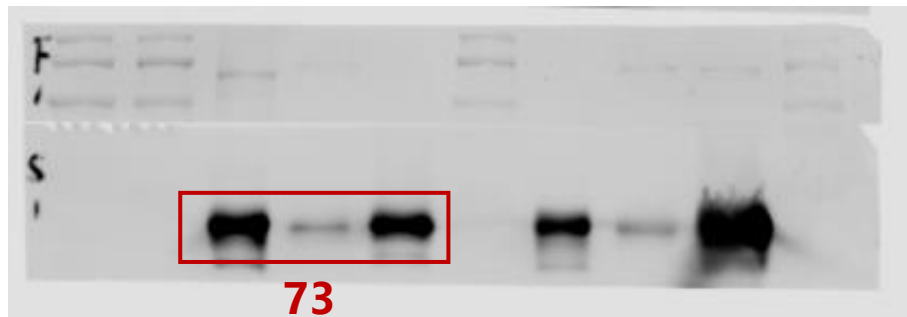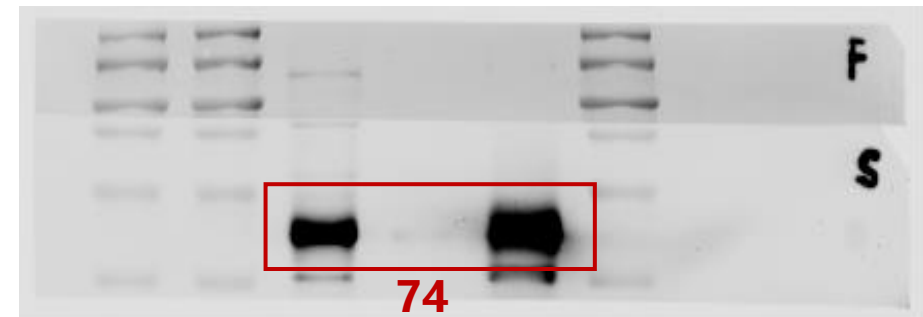

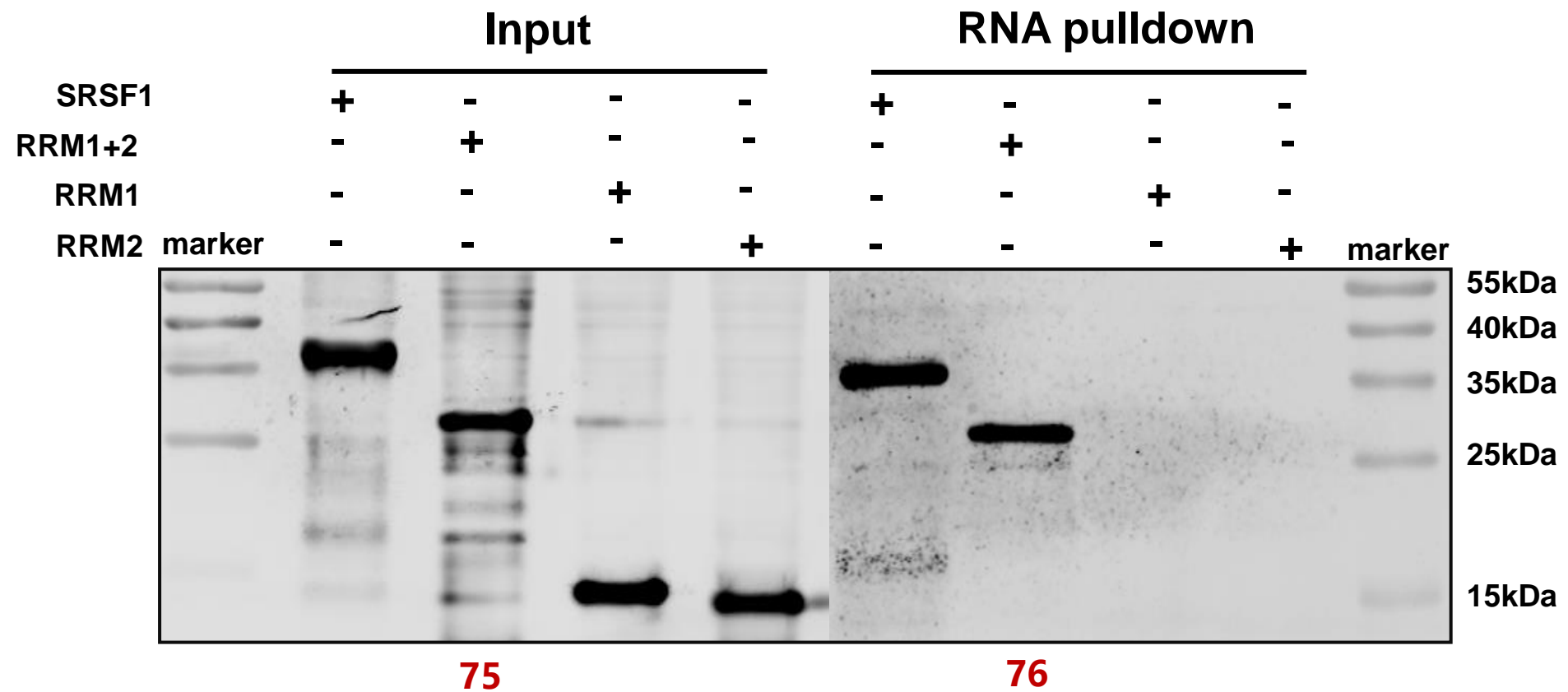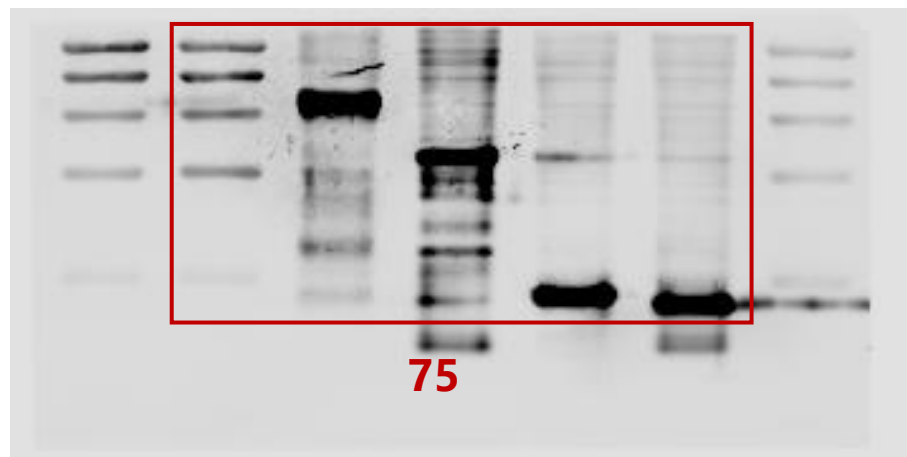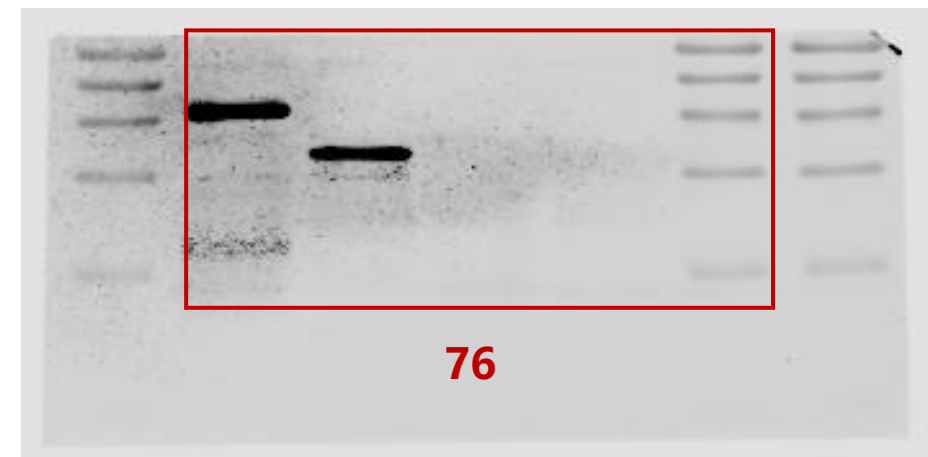

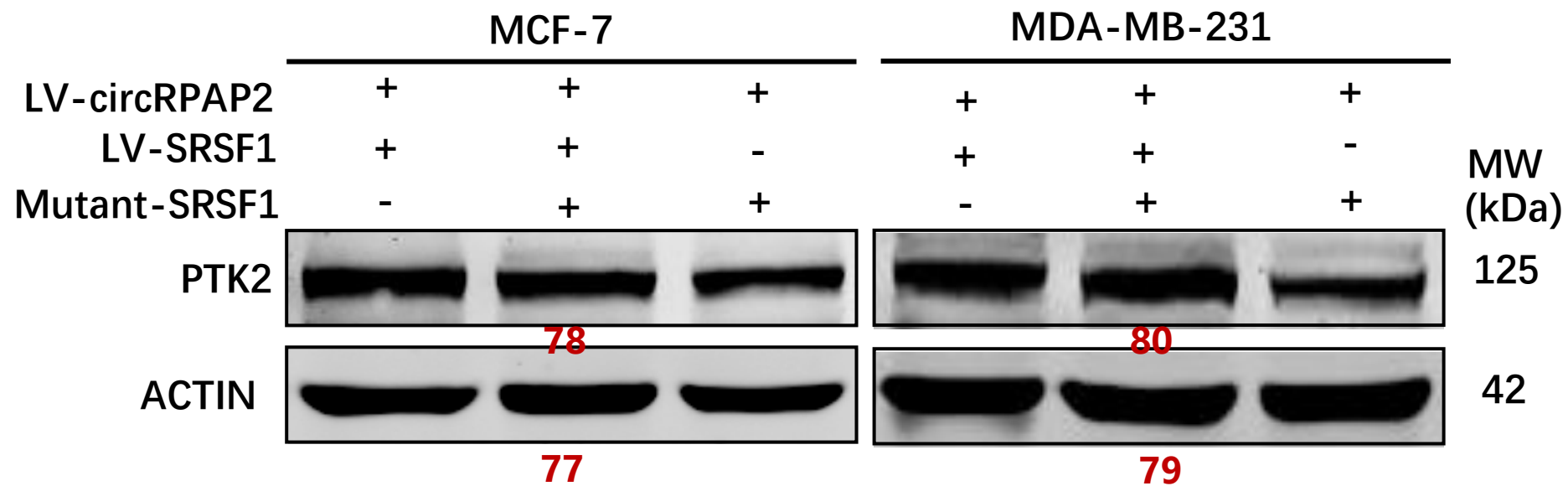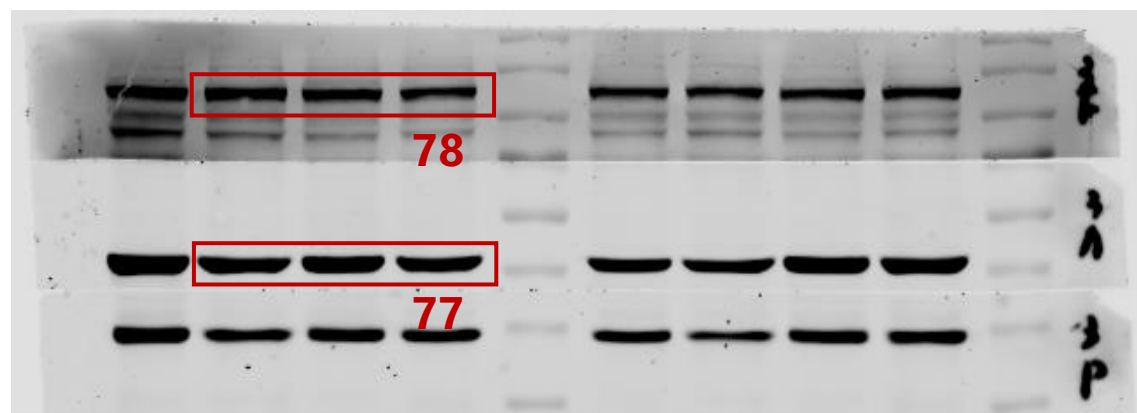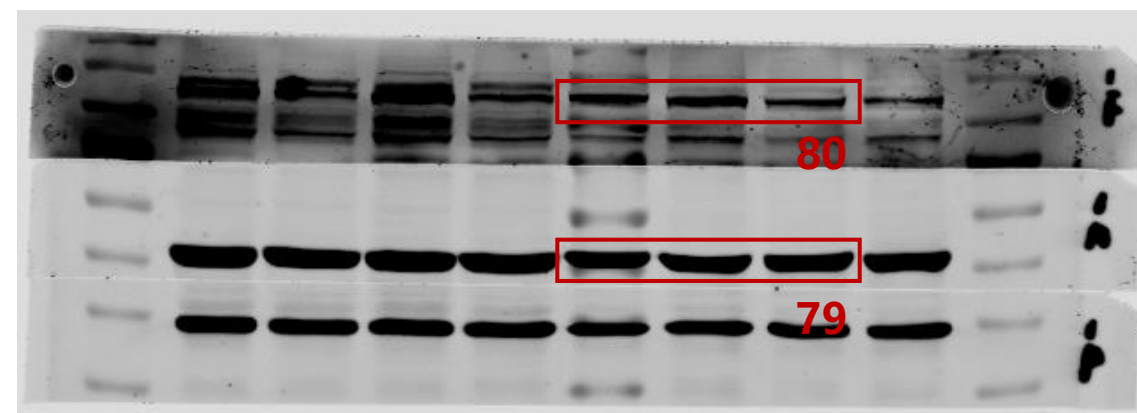

Supplement: Supplementary file 6 — Original Data File [file 41420_2022_965_MOESM6_ESM.pdf]
