# Supplementary material for: CircRPAP2 regulates the alternative splicing of PTK2 by binding to SRSF1 in breast cancer
Source: Cell Death Discov. 2022 Apr 2;8:152. doi: 10.1038/s41420-022-00965-y (PMC8976847; doi:10.1038/s41420-022-00965-y)
Supplement: Supplementary file 5 — Figure legends for supplemental figures [file 41420_2022_965_MOESM5_ESM.docx]

**Figure legends for supplemental figures**

**Figure S1.** **Effect of *si2-circRPAP2* and *si3-circRPAP2*.**

**(a)** Relative expression level of *circRPAP2* was confirmed by qRT-PCR in BC cells transfected with si-NC, *si2-circRPAP2* or *si3-circRPAP2*. **(b-e)** Effect of si2-circRPAP2 and si3-circRPAP2 on BC cell proliferation as determined by MTT assays. (**f)** Effect of si2-circRPAP2 and si3-circRPAP2 on BC cell proliferation as determined by western blotting. (* p < 0.05, **p < 0.01, ns: no significance).

**Figure S2. No significant effect of SRSF1 on *circRPAP2* and *RPAP2* expression level.**

**(a and b)** Relative expression level of *circRPAP2* was confirmed by qRT-PCR in BC cells transfected with si-NC, *si-SRSF1*, LV-vector, or *LV-SRSF1*. **(c and d)** Relative expression level of *RPAP2* were confirmed by qRT-PCR in BC cells transfected with si-NC, *si-SRSF1*, LV-vector, or *LV-SRSF1*. (ns: no significance).
